# Transcriptome profiling reveals the impact of various levels of biochar application on the growth of flue-cured tobacco plants

**DOI:** 10.1186/s12870-024-05321-z

**Published:** 2024-07-10

**Authors:** Yingfen Yang, Waqar Ahmed, Gang Wang, Chenghu Ye, Shichen Li, Meiwei Zhao, Jinhao Zhang, Junjie Wang, Saleh H. Salmen, Lianzhang Wu, Zhengxiong Zhao

**Affiliations:** 1 Yunnan Agricultural University, Kunming, Yunnan, 650201 China; 2 Jiangsu Key Laboratory for Bioresources of Saline Soils, Yancheng Teachers University, Yancheng, 224007 China; 3 Yunnan Revert Medical and Biotechnology Co., Ltd, Kunming, Yunnan, 65021 China; 4 Department of Botany and Microbiology, College of Science, King Saud University, Riyadh, 11451 Saudi Arabia; 5 Nujiang Green Spice Industry Research Institute, Lushui, Yunnan, 673200 China

**Keywords:** *Nicotiana tabacum*, Biochar, Plant physiology, Enzyme activity, Transcriptome

## Abstract

**Background:**

Biochar, a carbon-rich source and natural growth stimulant, is usually produced by the pyrolysis of agricultural biomass. It is widely used to enhance plant growth, enzyme activity, and crop productivity. However, there are no conclusive studies on how different levels of biochar application influence these systems.

**Methods and results:**

The present study elucidated the dose-dependent effects of biochar application on the physiological performance, enzyme activity, and dry matter accumulation of tobacco plants via field experiments. In addition, transcriptome analysis was performed on 60-day-old (early growth stage) and 100-day-old (late growth stage) tobacco leaves to determine the changes in transcript levels at the molecular level under various biochar application levels (0, 600, and 1800 kg/ha). The results demonstrated that optimum biochar application enhances plant growth, regulates enzymatic activity, and promotes biomass accumulation in tobacco plants, while higher biochar doses had adverse effects. Furthermore, transcriptome analysis revealed a total of 6561 differentially expressed genes (DEGs) that were up- or down-regulated in the groupwise comparison under different treatments. KEGG pathways analysis demonstrated that carbon fixation in photosynthetic organisms (ko00710), photosynthesis (ko00195), and starch and sucrose metabolism (ko00500) pathways were significantly up-regulated under the optimal biochar dosage (600 kg/ha) and down-regulated under the higher biochar dosage (1800 kg/ha).

**Conclusion:**

Collectively, these results indicate that biochar application at an optimal rate (600 kg/ha) could positively affect photosynthesis and carbon fixation, which in turn increased the synthesis and accumulation of sucrose and starch, thus promoting the growth and dry matter accumulation of tobacco plants. However, a higher biochar dosage (1800 kg/ha) disturbs the crucial source-sink balance of organic compounds and inhibits the growth of tobacco plants.

**Supplementary Information:**

The online version contains supplementary material available at 10.1186/s12870-024-05321-z.

## Introduction

Tobacco (*Nicotiana tabacum*) is an economically important industrial crop worldwide, including China [1]. China is among the world’s largest tobacco producers, with an annual production of 2.1 million tons of tobacco leaves. It is widely cultivated in south-central regions such as Yunnan Province due to its optimum climate for tobacco cultivation [2]. Yunnan contributes to half of China’s tobacco production and is known for its unique flavor and exotic aroma [2, 3]. To sustain higher production, farmers tend to apply large quantities of chemical fertilizers to promote growth and obtain maximum yields [1]. Although chemical fertilizers are readily taken up by plants, they have various shortcomings, such as nutrient loss, soil and water contamination, disturbance in soil pH and loss of beneficial microbial communities [4, 5]. Additionally, they are not an economically sustainable solution for crop production [6]. Therefore, much attention is being given to the use of environmentally friendly and cost-effective organic fertilizers.

Biochar (BC) is a naturally derived bio-stimulant that can profoundly enhance plant growth and improve soil fertility and stress resistance [7–9]. BC is derived from organic waste, crop residue, animal or poultry manure, etc., by heating at high temperatures (300–700 °C) in an oxygen-deficient environment in a process known as pyrolysis [10–12]. It is a rich carbon source, typically containing 70–80% carbon by weight. However, its nutrient content, including macro and micronutrients, can vary significantly depending on the feedstock used and the pyrolysis conditions [13]. The structure of BC is highly porous, with a large surface area and greater affinity toward charged molecules [14]. These properties contribute to its high adsorption and cation exchange capacity [15]. Therefore, in recent years, BC has been used for carbon sequestration, emission reduction and soil quality improvement [16]. Additionally, it is known that BC amendments improve microbial biomass and increase soil organic matter, aeration, water-holding capacity, and microbial activity [17–19].

In recent years, with the dramatic increase in crop production, the quantity of crop residue has also increased significantly [20]. Most crop residue is returned to the soil through management practices, but it has little or no impact, causes long-term soil acidification and promotes pest attack and disease [21]. In addition, crop residues are burned, which wastes natural resources and causes environmental pollution and human health concerns [3]. China produces 2 million tons of tobacco waste annually [22], and tobacco stems comprise 25–30% of the remaining waste after leaf collection. Therefore, converting the leftover tobacco stem into BC is an innovative and effective way to integrate natural resources into tobacco production.

The dosage of BC applied should be suitable according to plant requirements and soil properties [23]. For instance, when 1–4% BC is used, soil fertility, nutrient uptake capacity, and crop production increase. However, applying more than 5% BC has a detrimental effect on soil fertility and hinders plant growth [24]. The optimal application of BC can enhance soil enzymatic activities, thereby improving soil fertility and crop growth [25]. Recently, Zheng and colleagues (2021) reported that tobacco straw-derived BC application at a rate of 0 to 10 g/kg dry soil increased most of the agronomic traits of plants, while 50 g/kg BC inhibited their growth. Moreover, the activities of soil enzymes such as acid phosphatase, invertase, and urease increased, while catalase activity either decreased or remained the same [26]. However, the underlying molecular mechanisms of BC-mediated growth promotion in plants are still highly elusive.

Transcriptome profiling offers a cost-effective and efficient strategy to unravel molecular mechanisms related to growth promotion under different treatments [27]. A study on Chinese cherries treated with tobacco straw BC revealed that genes related to phytohormones such as abscisic acid, indole acetic acid and gibberellins were regulated, and photosynthesis was also promoted [22]. Several studies have reported the growth-promoting effect of BC at lower dosages and the inhibition of growth at higher dosages [28], but the underlying molecular mechanisms are poorly understood. Therefore, transcriptomics may allow us to study key genetic factors and pathways involved in the growth promotion of tobacco under different BC application rates. The present study investigated the effects of various dosages of tobacco stem-derived BC on growth promotion, enzyme activity, and biomass accumulation of tobacco plants. Further transcriptome profiling evaluated the differentially expressed genes enriched in growth-related pathways. Our findings provide mechanistic knowledge regarding important genetic elements involved in key pathways, such as photosynthesis, carbon fixation, and sucrose and starch metabolism, which are involved in growth promotion and inhibition under different BC application rates.

## Materials and methods

### Experimental site conditions, plant material, and morphological analysis of tobacco stem biochar

A field experiment was conducted with the tobacco cultivar Yunyan87 during the growing season from April to September 2021 at Jiangpo town (N25°15’, E101°40’), Mouding County, Chuxiong Prefecture, which is situated in the subtropical monsoon zone. The climatic conditions at the experimental site were as follows: 1736 m of altitude, annual temperature (average: 15.8 ℃), annual rainfall of 872 mm, a 238-day frost-free period, and 2359 h of annual sunshine. Seeds of flue-cured tobacco cultivar Yunyan87 were purchased from Yuxi Zhong Yan Seed Co. Ltd. (Yunnan, China). Tobacco seeds were sown in polystyrene trays (162 wells) contained mixed nursery medium (perlite, vermiculite, and turf; 3:3:4) as a substrate and placed under greenhouse [29]. Tobacco stems left over after harvesting tobacco leaves were used as raw material for the preparation of biochar. For collection of tobacco stem, the tobacco plants were topped completely, and tobacco stem biochar (BC) was prepared by pyrolysis at 450 ℃ for 4 h [7]. Morphological analysis of BC was performed using scanning electron microscopy (SEM; FlexSEM1000) according to the methodology of Nafees, et al. [30]. The physicochemical properties of soil and BC used in this experiment are summarized in Table 1.

**Table 1:** Physicochemical properties of soil and biochar used in this study

Soil	
pH	6.81
Organic carbon	17.83 g/kg
Total nitrogen	1.95 g/kg
Available nitrogen	122.9 g/kg
Available phosphorus	7.81 mg/kg
Available potassium	151.2 mg/kg
**Biochar**	
pH	10.16
Total carbon	57.83%
Total nitrogen	2.05%
Total phosphorus	1.24%
Total potassium	3.65%
Total cation exchange	12.07 cmol/kg
Electrical conductivity	3.68 µS/cm
Bulk density	0.23 g/cm^3^
Ash contents	28.9%

### Field experiment

In April 2021, field preparation, such as fertilizer application, ridge raising, and BC amendment, was performed. Biochar and fertilizer (base fertilizer) were thoroughly mixed into the soil in planting holes before transplanting seedlings. After field preparation, tobacco seedlings (50 days old) were transplanted in the planting holes on the ridges with plant × row spacings (60 cm × 120 cm) [28]. The experiment was conducted under five treatments: no BC (0 kg/ha; CK), 600 kg/ha BC (A1), 900 kg/ha BC (A2), 1200 kg/ha BC (A3), and 1800 kg/ha BC (A4). In addition, to avoid nutrient deficiency, base and top fertilizer was applied in the form of compound fertilizer (N-P_2_O_5_-K_2_O; 15-15-18) at 600 kg/ha according to the N: P:K ratio (1:2:2.5). Before seedling transplantation, 70% of the base fertilizer was applied, and within 35 days after seedling transplantation, the remaining 30% of the fertilizer was applied as top fertilizer [1]. All possible combinations of BC treatment levels (0 g, 40 g, 60 g, 80 g, and 120 g per hole or plant) were prepared and mixed thoroughly into the soil in the planting holes before transplanting the seedlings. In addition, field management activities were conducted following the national norms of China’s tobacco industry [31]. The experiment was performed using a randomized complete block design with a total of 15 plots and 3 plots (Plot size = 6 m × 7.4 m and 65 plants per plot) as replicates per treatment.

### Plant agronomic trait measurement

Tobacco plants were treated by first-flower topping, usually retaining about 18 to 22 leaves, depending on the plant’s growth. The plant agronomic traits, such as plant height, leaf number, stem circumference, maximum leaf length, maximum leaf breadth, and other pertinent data, were measured in accordance with the China Tobacco Industry Standard YC/T 142–1988 [7]. All the data were recorded at 60 d (late peak growth stage) and 100 d (lower leaf maturity stage) from each plot (10 plants/plot). The leaf area was calculated using the following formula [7]:

$$\eqalign{{\rm{leaf}}\,{\rm{area}}\,{\rm{ = }}\, & {\rm{maximum\,leaf\,length}} \cr & {\rm{ \times maximum\,leaf\,width \times 0}}{\rm{.6345}}{\rm{.}} \cr}$$

### Assessment of enzyme activity in tobacco leaves

Three tobacco plants were randomly selected at 60 d and 100 d after transplanting from each plot per treatment, and the 10th and 11th leaves from top to bottom were sampled to assess enzyme activity [32]. The sampled leaves were placed in test tubes, quickly transferred to liquid nitrogen and processed in the laboratory for further analysis. The activities of enzymes, including nitrate reductase (NR), glutamine synthetase (GS), sucrose synthase (SS), and sucrose phosphate synthase (SPS), were recorded by using kits from Suzhou Grace Biotechnology Co., Ltd., following the manufacturer’s instructions.

### Biomass accumulation calculation

At 60 and 100 days after transplantation, the biomass accumulation (g/plant) of different parts (roots, stems, and leaves) of the flue-cured tobacco plants under different treatments was recorded [33]. Five tobacco plants from each plot per treatment were removed and divided into three parts (roots, stems, and leaves). The plant parts were incubated at 105 ℃ for 30 min and then dried at 80 ℃ for 48 h. The dry weight of each plant part was measured, and biomass accumulation was calculated using the following formula [7]:

$$\eqalign{{\rm{Whole\,plant\,}} & {\rm{biomass\,accumulation}}\left( {{\rm{g/plant}}} \right){\rm{ = }} \cr & \sum {\left( {{\rm{biomass\,accumulation\,in\,leaves}}\,{\rm{ + }}\,{\rm{stems}}\,{\rm{ + }}\,{\rm{roots}}} \right)} {\rm{}} \cr}$$

### Transcriptome profiling

#### Sample collection

Middle leaf samples (3 samples per treatment) of tobacco plants treated with 0 kg/ha, 600 kg/ha and 1800 kg/ha BC were collected at 60 d and 100 d post-transplantation. The samples collected for RNA-seq analysis from 0 kg/ha, 600 kg/ha, and 1800 kg/ha BC were labeled PCK, PA1, and PA4, respectively, at 60 d post-transplantation and MCK, MA1, and MA4, respectively, at 100 d post-transplantation.

#### RNA purification, library construction, and sequencing

Plant RNA was extracted from 18 samples using a TRIzol Plant RNA Extraction Kit (Invitrogen Life Technologies, USA). The quality and quantity of RNA were evaluated using an Agilent 2100 Bioanalyzer (Agilent Technologies, Santa Clara, CA, USA) [34]. cDNA libraries were constructed using the NEBNext Ultra RNA Library Prep Kit for Illumina (New England Biolabs, USA) according to the manufacturer’s protocols. PCR amplification was performed using cDNA [35], and the obtained PCR products were sequenced on an Illumina HiSeqTM 4000 (Gene Denovo Biotechnology Co., Guangzhou, China).

#### Sequence mapping and gene expression analysis

To conduct a more in-depth study, we produced high-quality clean reads by applying the fastp (version 0.18.0) tool [36]. This involved filtering out reads that contained adapters, reads with more than 10% unknown nucleotides, and low-quality reads with more than 50% low-quality bases (with a Q value of 20) [37]. The collected clean reads were subsequently processed and aligned to the reference genome of *Nicotiana tabacum* [38] using HISAT2 version 2.4 [39], and gene expression was estimated as FPKM values via RSEM [40].

#### Differential gene (DEG) enrichment analysis

The expression levels of DEGs between samples and groups were calculated through the FPKM method using DESeq2 [41] following the methodology of Robinson [42]. DEGs were defined as genes with an adjusted p-value (q-value) ≤ 0.05 and an absolute value of log2-fold change (FC) ≥ 2 [34]. Gene enrichment was studied by conducting Gene Ontology (GO) [43] and Kyoto Encyclopedia of Genes and Genomes (KEGG) [44] enrichment analyses. A significance level (*p* ≤ 0.05) was used to determine the presence of significantly enriched DEGs for GO terms and KEGG pathways.

#### qRT‒PCR amplification for validation of RNA-seq data

To validate the RNA-seq data, qRT‒PCR was performed with SYBR Green Master Mix (Roche, Basel, Switzerland) as described previously [3]. Total RNA was extracted as described above, and cDNA was synthesized using the cDNA synthesis kit TIANGEN^®^ Biotech (Beijing, China) Co., Ltd. In total, 12 DEGs from the RNA-seq data were selected, and the tobacco *Actin* gene was used as a standard. The gene expression level was quantified using the 2^−△△Ct^ method [34] with three biological replicates. The gene-specific primers used in this study are listed in Table S1.

### Statistical analysis

The data related to enzyme activities and biomass accumulation were analyzed through ANOVA in SPSS (V. 25. IBM, USA), represented as means standard errors (± SE), and were considered to be significantly different if *p* < 0.05 according to the least significant difference test [45]. The qRT‒PCR data were subjected to Student’s t-test in GraphPad Prism (version 8.0, San Diego, California, USA).

## Results

### Physicochemical and morphological properties of biochar

**Fig. 1:**
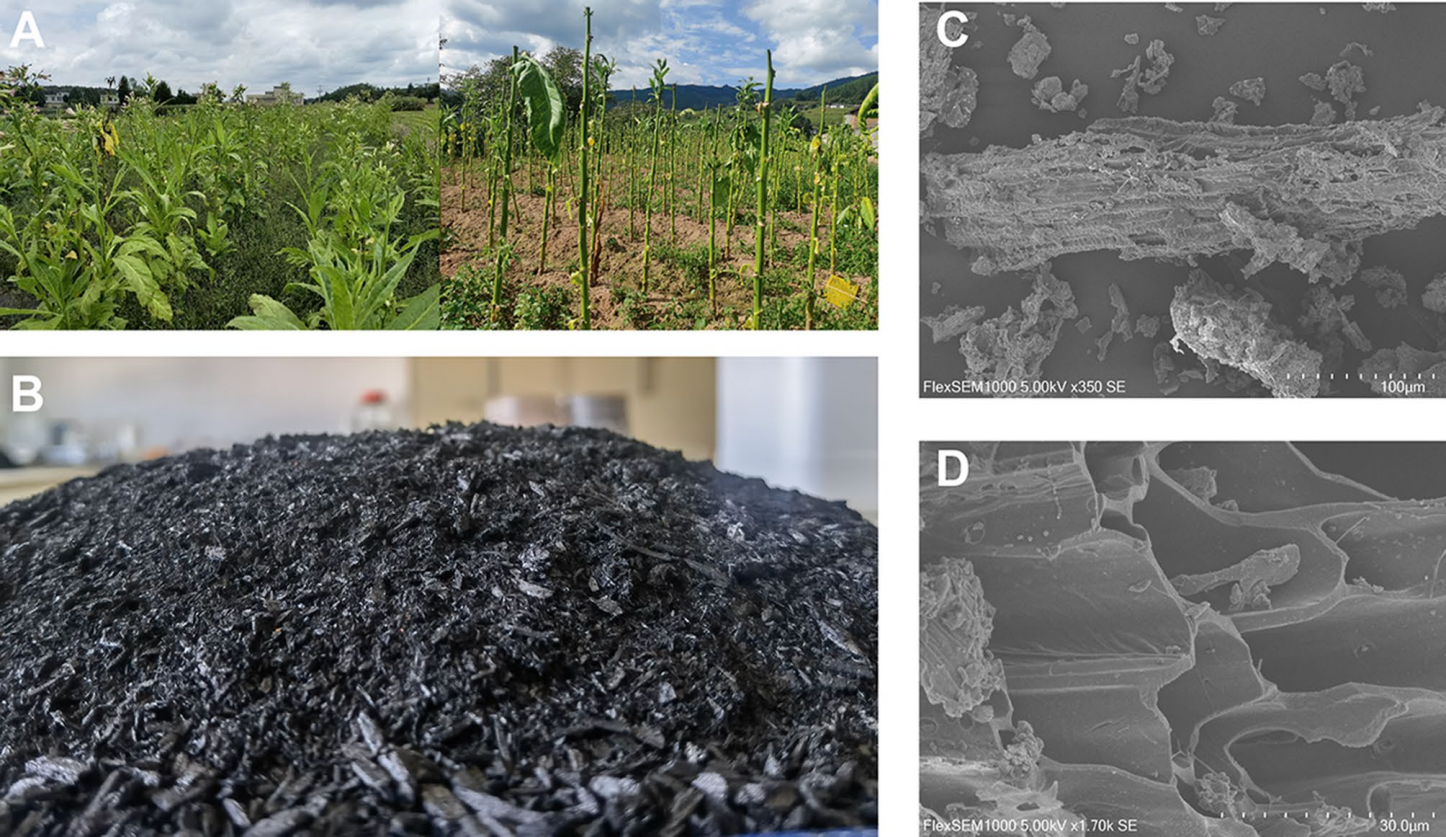
Morphological properties of biochar obtained by pyrolysis of tobacco stems. (**A**) Tobacco stems in the field after leaf picking. (**B**) Biochar was obtained through pyrolysis at 450 ℃ for 4 h. (**C**-**D**) Scanning electron microscopy of the obtained biochar at 100 μm and 30 μm, respectively

The tobacco stems remaining after picking tobacco leaves were used to prepare biochar (BC) (Fig. 1). Furthermore, BC scanning electron microscopy (SEM) analysis revealed a porous structure and irregular shape, which is appropriate for providing habitat for soil microbes and water storage (Fig. 1).

### Biochar soil amendments affect tobacco plant growth

**Fig. 2:**
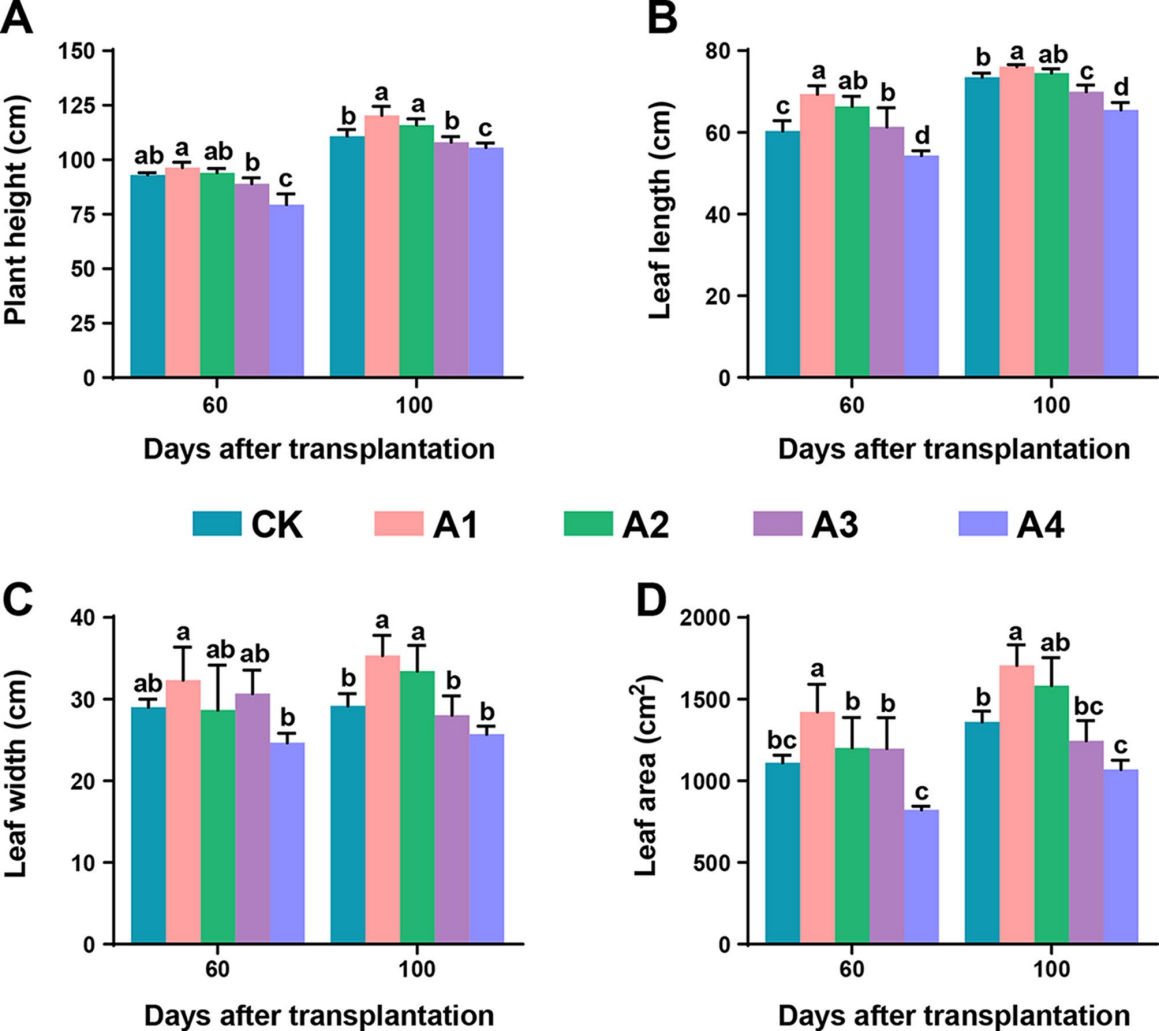
Effects of different doses of biochar on plant physiological traits. (**A**) Plant height, (**B**) leaf length, (**C**) leaf width, and (**D**) leaf area at 60 days and 100 days after transplantation. Here, CK, no biochar (0 kg/ha); A1, 600 kg/ha biochar; A2, 900 kg/ha biochar; A3, 1200 kg/ha biochar; and A4, 1800 kg/ha biochar. The significant differences among treatments are shown by different lowercase letters on the error bars according to the least significant difference test (LSD, *p* < 0.05)

The effect of varying BC dosages on several growth traits of tobacco plants was assessed. The height of the tobacco plants was more significant at both 60 d and 100 d after transplantation when 600 kg/ha (PA1 and MA1) was applied than in the control and other treatments (Fig. 2). A similar trend was observed for other traits; the leaf length, width, and area were greater in A1 and A2 at 60 d and 100 d after transplantation than in CK, A3 and A4. However, these traits in A3 and A4 (1200 and 1800 kg/ha) were either lower than those in CK or not significantly different. A relatively high BC dosage had an inverse relationship with the growth traits of tobacco plants, while the application of 600 kg/ha and 900 kg/ha BC had positive effects on both the early and late growth stages.

### Modulation of enzymatic activities under biochar application

**Fig. 3:**
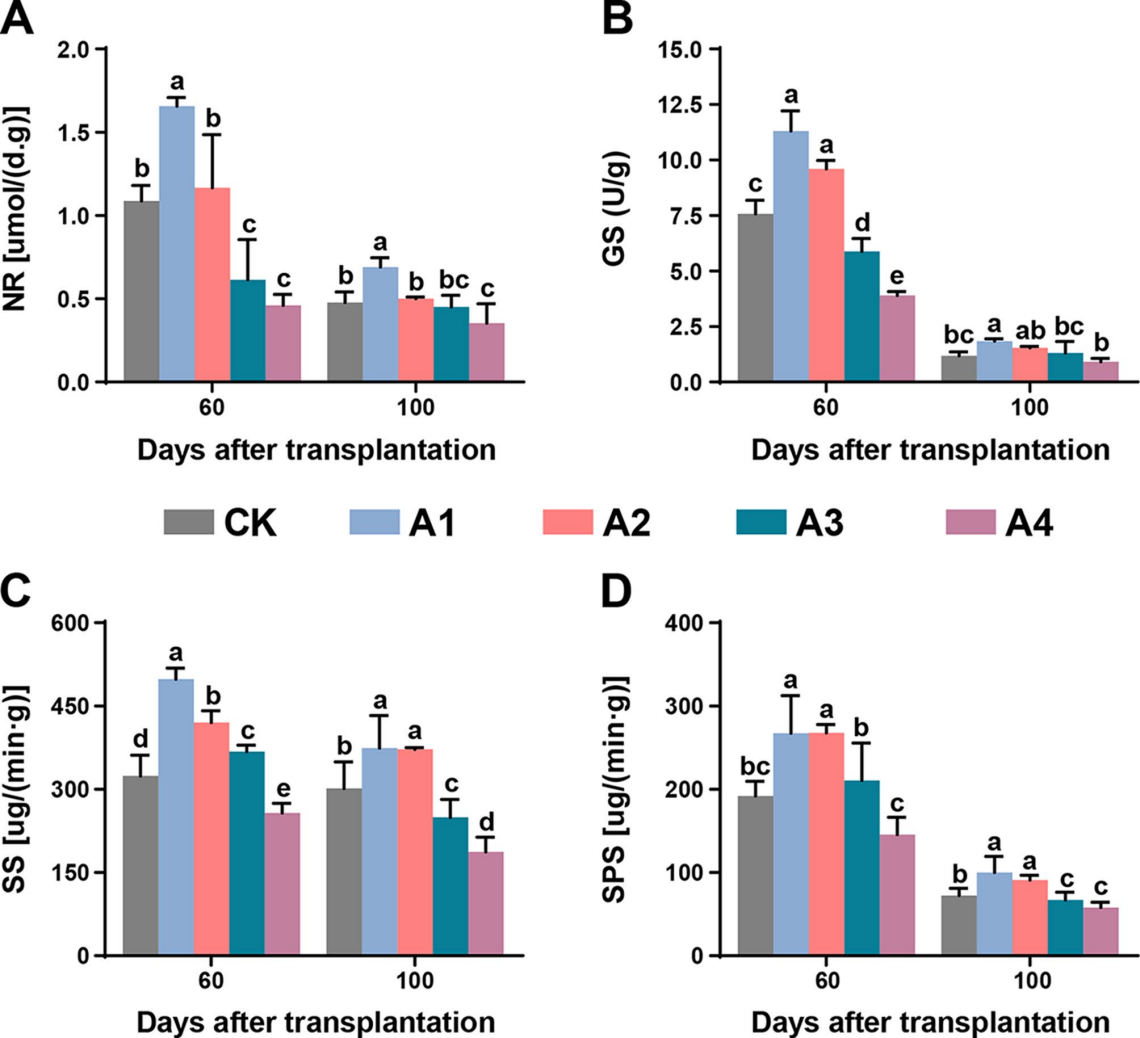
Effect of different biochar application levels on the activity of plant enzymes. (**A**) Nitrate reductase, (**B**) glutamine synthetase, (**C**) sucrose synthase, and (**D**) sucrose phosphate synthase at 60 days and 100 days after transplantation. The significant differences among treatments are shown by different lowercase letters on the error bars according to the least significant difference test (LSD, *p* < 0.05). Here, CK, no biochar (0 kg/ha); A1, 600 kg/ha biochar; A2, 900 kg/ha biochar; A3, 1200 kg/ha biochar; and A4, 1800 kg/ha biochar

BC application may affect plant enzymatic activities to a certain extent. Therefore, the effects of different doses of BC on the activity of four enzymes, nitrate reductase (NR), sucrose synthase (SS), glutamine synthetase (GS), and sucrose phosphate synthase (SPS), were investigated. Generally, the enzyme activity under BC treatment was more significant at 60 d than at 100 d. The activity of all four enzymes was greater in A1 and A2 than in CK and the other treatments at both time points except for SS and SPS, where both A1 and A2 were not significantly different at 100 d after transplantation. The levels of glutamine synthetase and sucrose synthase (SS) significantly differed among the different treatments, with the order from highest to lowest being A1 > A2 > CK > A3 > A4 and A1 > A2 > A3 > CK > A4, respectively (Fig. 3). Overall, the activity of all four enzymes increased to varying degrees with the application of 600 kg/ha and 900 kg/ha BC. The effect was non-significant at 1200 kg/ha and 1800 kg/ha dosage of BC, which reduced the activity of all enzymes. Therefore, a high dosage of BC can negatively impact the optimal functioning of the plant metabolic machinery.

### Biochar application enhances dry matter accumulation

**Fig. 4:**
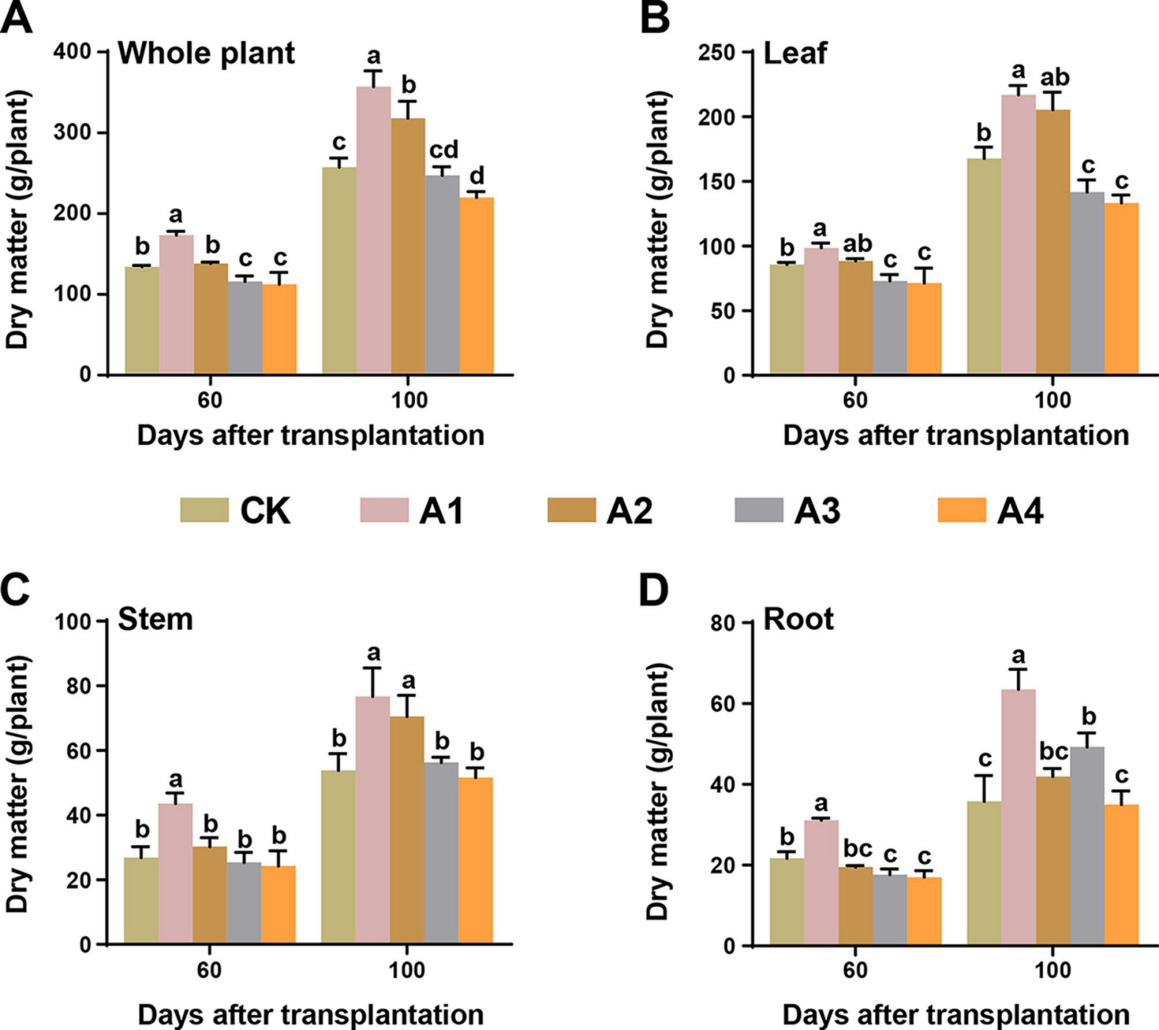
Effect of different biochar application rates on the accumulation of dry matter. (**A**) Whole plant, (**B**) leaf, (**C**) stem, and (**D**) root at 60 days and 100 days after transplantation. Here, CK, no biochar (0 kg/ha); A1, 600 kg/ha biochar; A2, 900 kg/ha biochar; A3, 1200 kg/ha biochar; and A4, 1800 kg/ha biochar. The significant differences among treatments are shown by different lowercase letters on the error bars according to the least significant difference test (LSD, *p* < 0.05)

At the optimum BC dosage, the addition of BC to the soil positively affected dry matter accumulation in the plants (Fig. 4). At 60 d after transplantation, the accumulation of dry matter in the whole plant and plant parts (leaf, stem and root) was significantly greater in the A1 treatment than in the CK and other treatments. Under the A2 treatment, the dry matter accumulation in whole plants and leaves was considerably greater than that under the CK, A3 and A4 treatments. Generally, the dry matter allocation among organs decreased in the following order: leaf > stem > root. At 100 d after transplantation, the dry matter accumulation was significantly greater in plants treated with A2 than in those treated with CK, A3 or A4. The dry matter accumulation in whole plants and leaves was significantly lower under the A3 and A4 treatments than under the CK treatment. The dry matter accumulation in the stems and roots under the A4 treatment was not significantly different from that under the MCK treatment. The highest amount of dry matter accumulation in the whole plant and plant parts occurred when 600 kg/ha BC was applied, followed by 900 kg/ha BC at both the early and late growth stages. However, excessive application (1200 kg/ha and 1800 kg/ha) of BC has a detrimental effect on the growth.

### Transcriptome sequencing and quality assessment

In total, 18 samples (3 treatments × 2 time points × 3 biological replicates per treatment) were sequenced to obtain 773,879,730 raw reads. After filtering the low-quality reads and adapter sequences, 767,963,260 clean reads were retained and mapped to the tobacco reference genome, of which 662,384,740 reads were mapped, accounting for 90.89–93.59% of the total raw reads, highlighting efficient read mapping. Among the mapped reads, 643,751,437 were mapped to unique positions, accounting for an average of 89.98%. Furthermore, the GC content of all the samples ranged between 42.81% and 46.94%, with an average of 43.77% per sample. The Q30% ranged from 92.9 to 94.11%, with an average of 93.42% for each sample (Table S2).

**Fig. 5:**
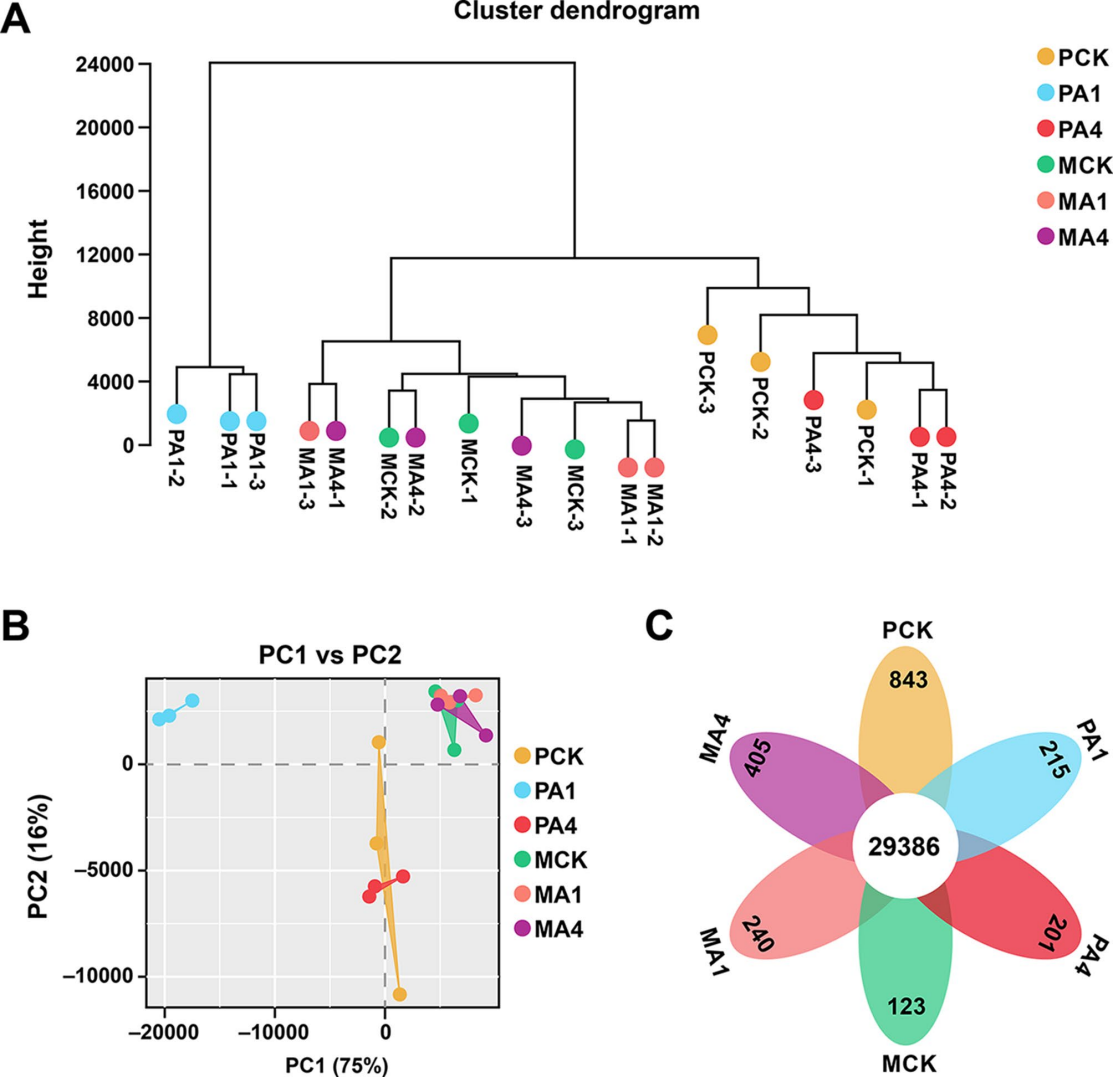
Differences in transcriptome sequences under different biochar application rates. (**A**) Sample clustering through a dendrogram. (**B**) Principal component analysis and (**C**) Venn diagram showing unique and commonly expressed genes in various treatment groups. Here, PCK, PA1, and PA4 represent the samples collected from plants treated with 0, 600, and 1800 kg/ha biochar, respectively, after 60 days post-transplantation. MCK, MA1, and MA4 represent the samples collected from plants treated with 0, 600, and 1800 kg/ha biochar, respectively, after 100 days post-transplantation

Sample clustering and principal component analysis (PCA) were performed to check the variations among the treatments (Fig. 5). The dendrogram highlights the clustering of different treatments; samples of PA1 cluster together, whereas PA4 is more related to PCK. Similarly, MCK, MA1, and MA4 were more similar to each other than the other treatments (Fig. 5A). PCA also confirmed the results from sample clustering, showing high dispersion among PA1, PA4 and PCK, while MA1, MA4 and MCK were closely clustered (Fig. 5B). The first two principal components accounted for 91% of the variability among the six groups in triplicate. The Pearson correlation test between different replicates showed that the gene expression level was consistent across the samples from other treatments (Figure S1). The high-quality reads, variability among various treatment groups and similarity within the treatment samples support the reliability of the data for further analysis.

### Identification of DEGs

**Fig. 6:**
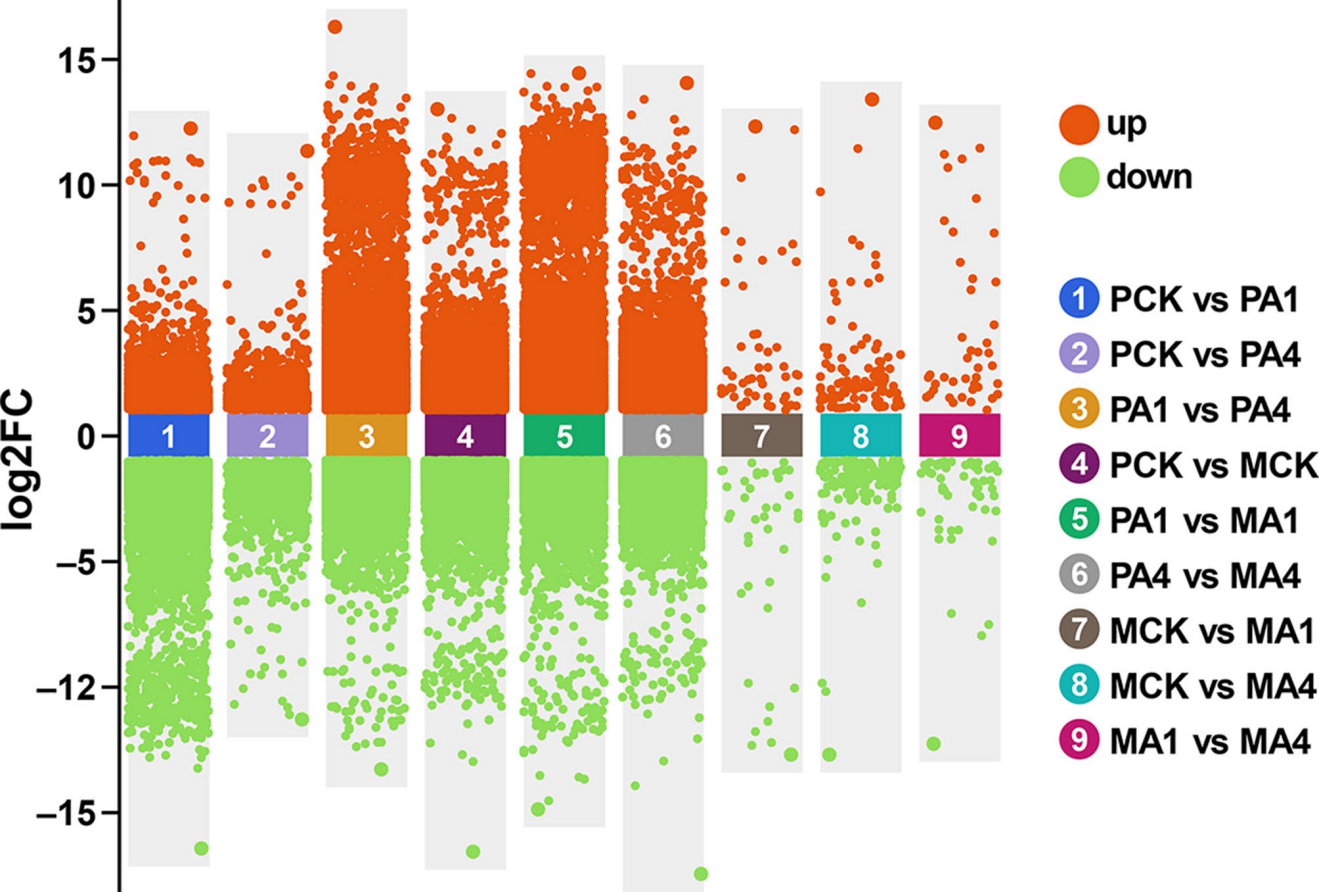
Differentially expressed genes analysis in groupwise comparisons under different biochar treatments. The up-regulated DEGs are located in the orange region, while the down-regulated DEGs are located in the green region. Here, PCK, PA1, and PA4 represent the samples collected from plants treated with 0, 600, and 1800 kg/ha biochar, respectively, after 60 days post-transplantation. MCK, MA1, and MA4 represent the samples collected from plants treated with 0, 600, and 1800 kg/ha biochar, respectively, after 100 days post-transplantation

In total, 31,413 expressed genes (EGs) were detected in all samples, where 29,386 EGs were common in all groups, while 843, 215, and 201 EGs were present in PCK, PA1, and PA4, respectively. Similarly, 123, 240, and 405 EGs were identified from MCK, MA1 and MA4, respectively (Fig. 5C). In the pairwise comparison, a total of 6561 DEGs (2417 up-regulated and 4144 downregulated) were detected in PCK vs. PA1, while 1503 DEGs (713 up-regulated and 790 downregulated) were present in PCK vs. PA4, which is relatively less than in PCK vs. PA1 (Fig. 6, Table S3). However, the maximum number of DEGs (13,568 DEGs: 8805 up-regulated and 4763 downregulated) was detected in PA1 vs. MA1, followed by PA1 vs. PA4 (12,048 DEGs: 6544 up-regulated and 5504 downregulated). The lowest number of DEGs was found in the MCK vs. MA1 comparison (93 DEGs: 53 up-regulated and 40 downregulated), followed by the MA1 vs. MA4 comparison (96 DEGs: 43 up-regulated and 53 downregulated) (Figure S2). Interestingly, from the groupwise comparison, it is evident that compared with the late growth stage (100 d), the 600 kg/ha BC application (A1) had a profound effect on gene regulation at the early growth stage (60 d). Furthermore, we randomly selected 12 DEGs from the enriched pathways to validate the RNA-seq data. The expression patterns of all 12 DEGs were similar to the RNA-seq results (Figure S3).

### Functional classification and annotation of DEGs

The DEGs in pairwise groups were characterized by their predicted functions through Gene Ontology (GO) annotations and Kyoto Encyclopedia of Genes and Genomes (KEGG) pathway analysis. According to the GO enrichment analysis, all DEGs were classified into three categories: biological process (BP), cellular component (CC), and molecular function (MF) (Figure S4). In PCK vs. PA1, a total of 37,809 DEGs were classified into 23, 12, and 19 BP, CC and MF categories, respectively. In the BP category, response to stimulus (GO:0050896), biological regulation (GO:0065007), single-organism process (GO:0044699), cellular process (GO:0009987) and metabolic process (GO:0008152) were annotated. The categories of binding (GO:0005488), transporter activity (GO:0005215), and catalytic activity (GO:0003824) and organelle part (GO:0044422), membrane (GO:0016020), membrane part (GO:0044425), organelle (GO:0043226), cell (GO:0005623), and cell part (GO:0044464) were enriched in MF and CC, respectively (Figure S4-A). In PCK vs. PA4 and PA1 vs. PA4, similar categories from BP, MF and CC were annotated for a total of 8351 and 69,707 DEGs, respectively, as PCK vs. PA1, except organelle-part and membrane part from CC in PCK vs. PA4 and organelle from CC in PA1 vs. PA4 (Figure S4-B and -C). A total of 328, 1182 and 356 DEGs from MCK vs. MA1, MCK vs. MA4 and MA1 vs. MA4, respectively, were commonly annotated to cellular process (GO:0009987), metabolic process (GO:0008152) and single-organism process (GO:0044699); catalytic activity (GO:0003824) and binding (GO:0005488); and cell part (GO:0044464), cell (GO:0005623), and organelle (GO:0043226) from BP, MF and CC, respectively (Figure S4-D, -E, and -F). DEGs from the comparison groups between the treatments PCK vs. MCK, PA1 vs. MA1 and PA4 vs. MA4 were commonly annotated to the top three categories: single-organism process, cellular process and metabolic process from BP; catalytic activity and binding from MF; and cell, cell part and organelle from CC (Figure S4-G, -H, and -I).

To further investigate the differentially expressed transcripts in response to BC application, KEGG annotations were performed (Figure S5). In PCK vs. PA1, carbon fixation in photosynthetic organisms (ko00710), photosynthesis (ko00195), carbon metabolism (ko01200), and fructose and mannose metabolism (ko00051) pathways were enriched (Figure S5-A). However, in PCK vs. PA4, plant-pathogen interactions (ko04626), metabolic pathways (ko01100), plant hormone signal transduction (ko04075), and biosynthesis of secondary metabolites (ko01110) were regulated (Figure S5-B). Protein processing in the endoplasmic reticulum (ko04141), photosynthesis (ko00195), plant-pathogen interactions (ko04626), carbon fixation in photosynthetic organisms (ko00710), glyoxylate and dicarboxylate metabolism (ko00630), endocytosis (ko04144), starch and sucrose metabolism (ko00500), carbon metabolism (ko01200), metabolic pathways (ko01100), and nitrogen metabolism (ko00910) were differentially regulated in PA1 vs. PA4 (Figure S5-C). In contrast, protein processing in the endoplasmic reticulum (ko04141) and mRNA surveillance pathway (ko03015) were annotated for DEGs from MCK vs. MA1 (Figure S5-D), while biosynthesis of secondary metabolites (ko01110), metabolic pathways (ko01100), and phenylpropanoid biosynthesis (ko00940) were regulated in MCK vs. MA4 (Figure S5-E). Furthermore, DEGs from MA1 vs. MA4 were annotated to the flavonoid biosynthesis pathway (ko00941), phenylpropanoid biosynthesis pathway (ko00940), and stilbenoid, diarylheptanoid and gingerol biosynthesis pathways (ko00945) (Figure S5-F). However, 2943, 2820 and 2257 DEGs between treatments in the comparison groups—PA1 vs. MA1, PA4 vs. MA4 and PCK vs. MCK—were annotated to carbon metabolism, photosynthesis and carbon fixation in photosynthetic organisms; metabolic pathways, biosynthesis of secondary metabolites and protein processing in the endoplasmic reticulum; and metabolic pathways, biosynthesis of secondary metabolites and the MAPK signaling pathway, respectively (Figure S5-G, -H, and -I).

### Pathway regulation in flue-cured tobacco by biochar application

**Fig. 7:**
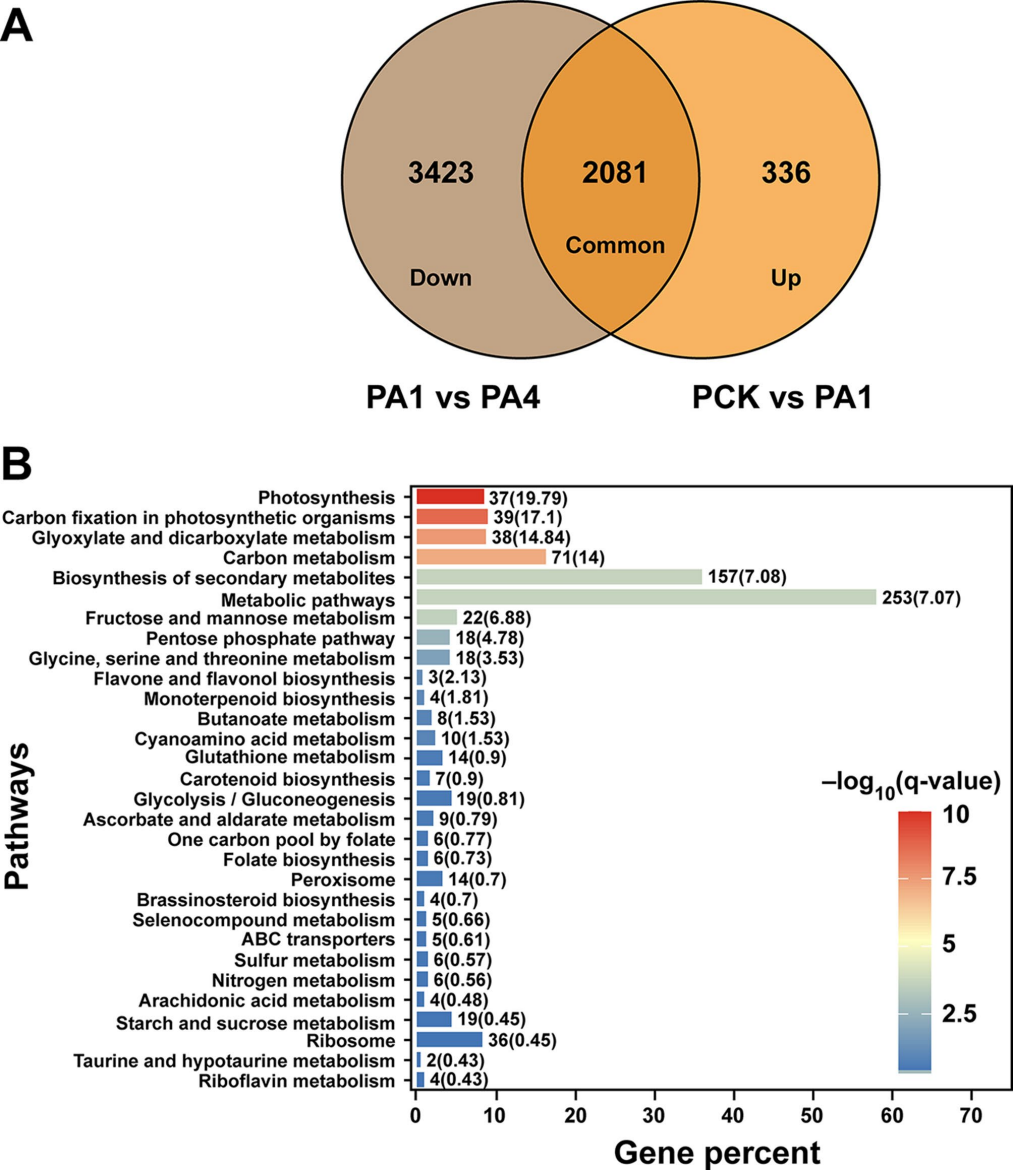
Classification of differentially expressed genes enriched in the two selected comparison groups. (**A**) Venn diagram showing common and unique DEGs in groupwise comparisons A1 vs. A4 and PCK vs. PA1 and (**B**) significantly enriched pathways from PCK vs. PA1 and PA1 vs. PA4

According to the groupwise comparison, the highest number of transcripts were differentially regulated between PCK vs. PA1 and PA1 vs. PA4 (Fig. 7). A Venn diagram shows the 2081 common DEGs from both comparison groups; 336 were up-regulated in PCK vs. PA1, and 3423 DEGs were downregulated in PA1 vs. PA4 (Fig. 7A). To gain in-depth insight, a collective KEGG analysis was performed, and the most enriched pathways were selected. Interestingly, pathways related to photosynthesis, carbon fixation in photosynthetic organisms and carbon metabolism were highly enriched (Fig. 7B).

#### Photosynthetic pathway

**Fig. 8:**
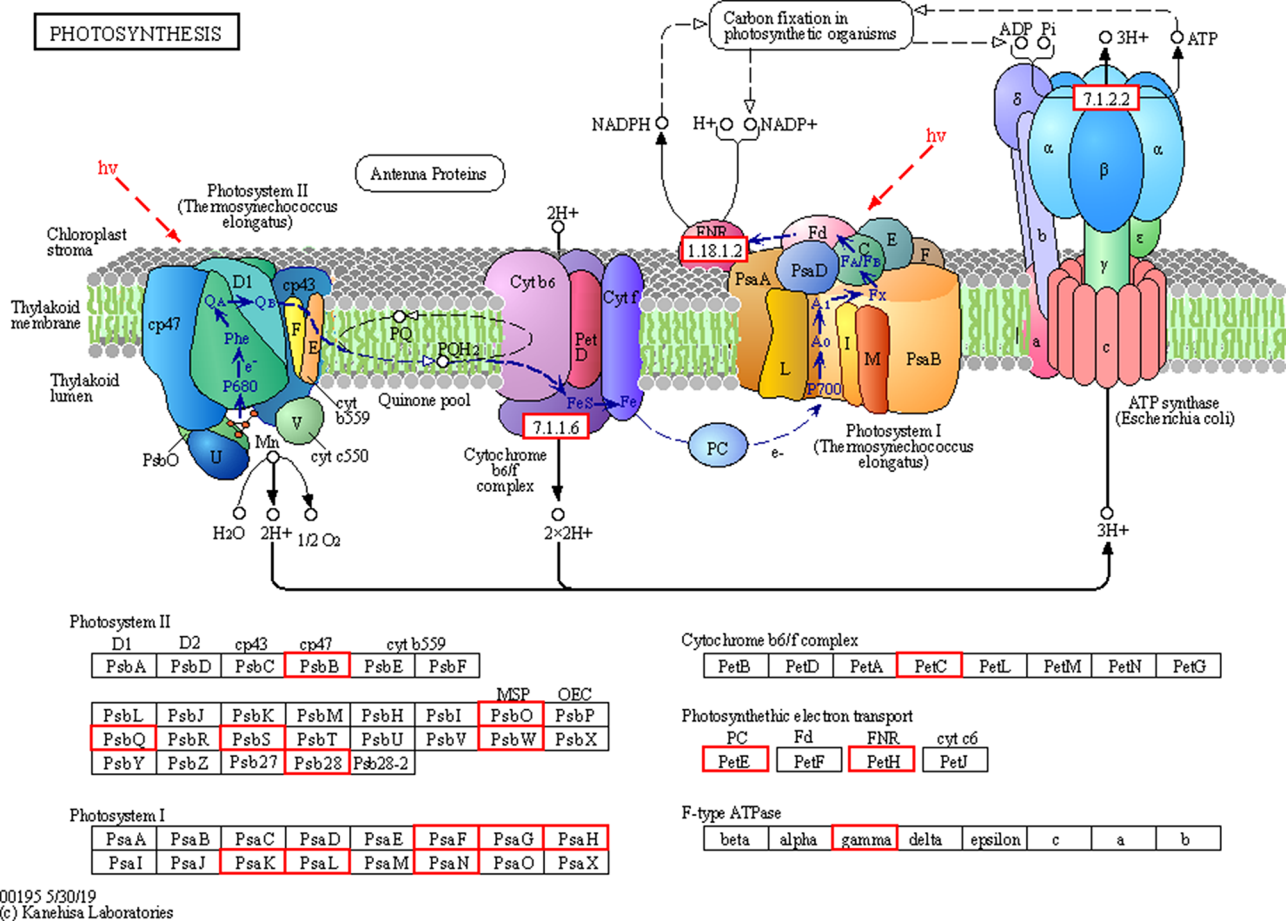
Photosynthesis in PCK vs. PA1 vs. PA4 highlights the role of differentially expressed genes involved in photosynthetic pathway regulation in response to biochar treatment. The red boxes show the up-regulated transcripts, which encode important structural and functional components of the photosynthetic machinery

Plants prepare their food through photosynthesis, and the processes of photosynthesis and growth are correlated. Many important genes encoding ferredoxin-NADP reductase (gene_677, gene_66199), cytochrome b6-f complex iron-sulfur subunit 2 (gene_17565), ATP synthase gamma chain (gene-59,741), and photosystem I reaction center subunit III (gene_32839, gene_46518) were up-regulated in plants treated with 600 kg/ha at 60 d compared to control plants (PCK vs. PA1). Other transcripts related to photosystems I and II were also significantly up-regulated in PCK vs. PA1 (Fig. 8). All these transcripts were downregulated in tobacco plants treated with 1800 kg/ha at 60 d compared to those treated with 600 kg/ha (PA1 vs. PA4). The DEGs enriched in the pathways are listed in Table S4 and Table S5.

#### DEGs involved in carbon fixation pathways

Following photosynthesis, plants fix CO_2_ through the Calvin cycle, which is a series of reactions catalyzed by several enzymes (Figure S6). In PCK vs. PA1, transcripts related to key enzymes involved in the Calvin cycle, such as ribulose bisphosphate carboxylase small chain (MSTRG.51,903, gene_14024, gene_44833, and gene_61363), fructose-1,6-bisphosphatase (gene_65990, gene_27987, gene_79620, gene_82004, gene_44694, gene_65991, and gene_67033), glyceraldehyde-3-phosphate dehydrogenase (gene_15058, gene_68511, gene_30765, and gene_66218), fructose-bisphosphate aldolase (gene_57160, gene_62167, gene_38021, gene_60848, and gene_73919), transketolase (gene_71228 and gene_10489), and sedoheptulose-1,7-bisphosphatase (gene_23845, gene_48874, and gene_42407), were significantly up-regulated. However, genes encoding phosphoenolpyruvate carboxylase (gene_61700 and gene_43822) were significantly downregulated in PA1 vs. PA4 (Table S6 and Table S7).

#### Modulation of starch and sucrose metabolism

**Fig. 9:**
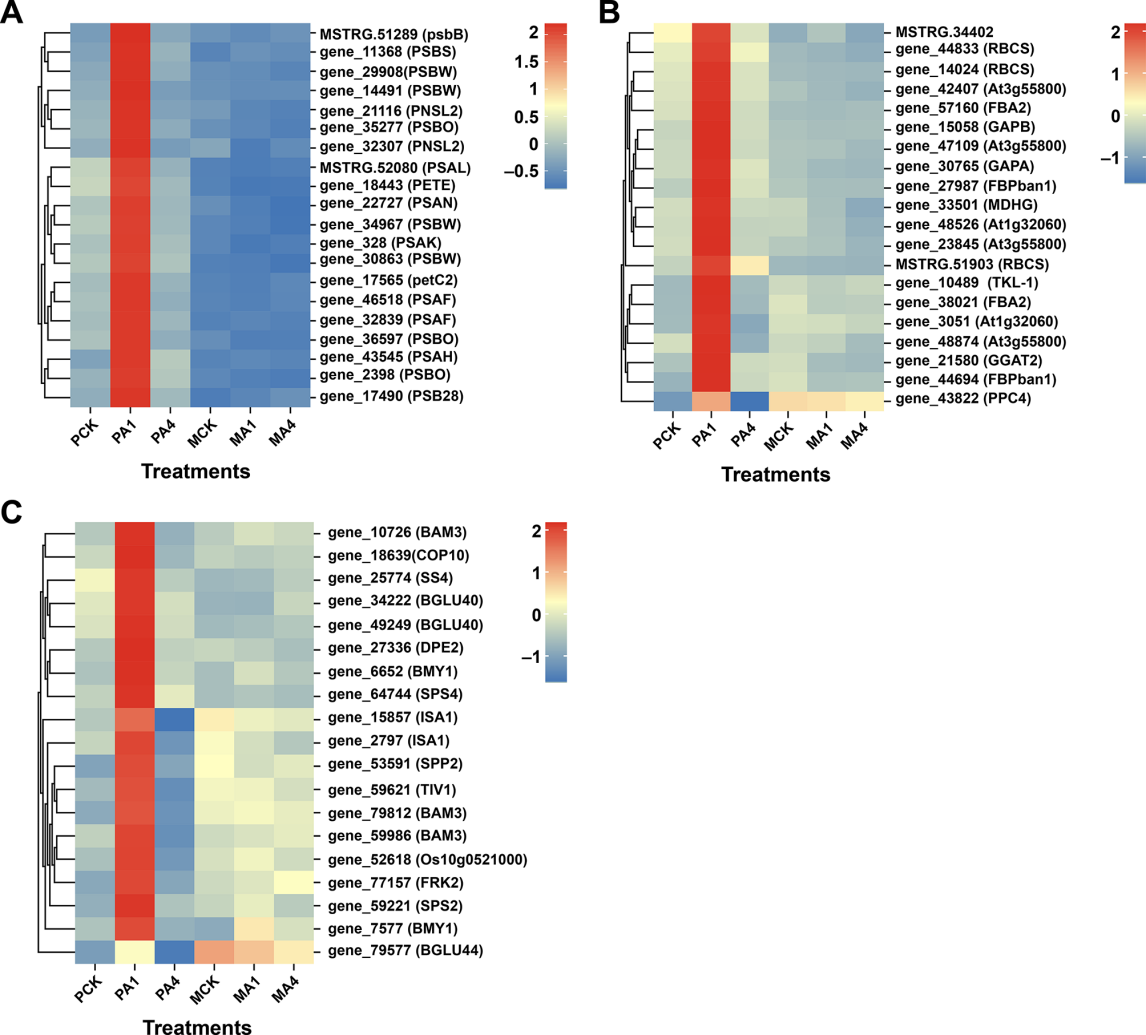
Heatmap showing the regulation of targeted genes under different treatments. (**A**) Photosynthetic pathway, (**B**) carbon fixation in photosynthetic organisms and (**C**) starch and sucrose metabolism. Red and blue correspond to up-regulated and downregulated transcripts, respectively

Carbon dioxide from the environment is efficiently assimilated at the end of the photosynthetic reaction, and the precursors in photosynthates are predominantly converted into starch and sucrose (Figure S7). Several transcripts related to starch and sucrose metabolism, such as beta-amylase (gene_10726, gene_79812, and gene_59986), sucrose-phosphate synthase (gene_64744 and gene_59221), sucrose-phosphatase (gene_53591), starch synthase (gene_25774), beta-glucosidase (gene_34222, gene_49249, and gene_79577) and others, were significantly up-regulated in PCK vs. PA1 but downregulated in PA1 vs. PA4. All regulated transcripts are listed in Table S8 and Table S9. Furthermore, a heatmap was constructed to investigate the regulation of selected DEGs across all treatments for photosynthesis, carbon fixation and sucrose and starch metabolism (Fig. 9). All transcripts were more highly expressed in PA1 than in PCK, PA4, MCK, MA1 and MA4. The upregulation of these transcripts highlights their role in promoting growth under BC application (600 kg/ha) (Fig. 9A-C).

## Discussion

Soil amendments with biochar (BC) improve crop growth and yield [7, 24]. The effects of biochar on plants are expected to vary with applied dosage and optimal dosages are required for normal growth [23]. Therefore, in the present study, different dosages of BC were applied to determine the effects on growth parameters, enzymatic activity and biomass accumulation in flue-cured tobacco plants. The application of 600 kg/ha BC increased growth parameters such as plant height, leaf length, leaf width and leaf area of flue-cured tobacco plants at 60 d and 100 d after transplantation. A higher dose of BC (1200 kg/ha and 1800 kg/ha) did not significantly improve the growth parameters at either time point; rather, a higher dosage of 1800 kg/ha negatively affected plant growth (Fig. 2). Therefore, it is evident that an optimal dosage of BC is conducive to plant growth, while a high dosage negatively regulates growth events. A negative linear relationship has been reported between increasing BC dosage and plant growth, as the growth of *Lolium multiflorum* was found to be inversely proportional to increasing BC dosage (0, 5, 10, 20, 50 ton/ha). It was reasoned that organic compounds leached from BC might be responsible for this adverse effect [46].

Similarly, the activities of four enzymes involved in key pathways in tobacco leaves were recorded under different BC dosages. Nitrate reductase is the first enzyme involved in the assimilation of N-NO_3_; thus, it plays a crucial role in plant development and nitrogen assimilation [47]. The NR activity was highest under the 600 kg/ha (A1) treatment at 60 d after transplantation, and increasing the BC dose reduced the enzymatic activity; however, at 100 d after transplantation, increasing the BC dose did not significantly affect the activity compared with that of the control plants. Another enzyme involved in nitrogen metabolism is glutamine synthetase, which plays a key role in nitrogen assimilation in plants and promotes plant development [48]. The GS activity in tobacco leaves was greatest at 60 d after transplantation when 600 kg/ha was applied, while it decreased with increasing BC dosage. An increased dosage of BC could interfere with the carbon-nitrogen exchange, which would hinder plant growth. Two key enzymes, sucrose synthase and sucrose phosphate synthase, which catalyze sucrose breakdown and synthesis, respectively, were also more strongly activated in PA1 than in the control and at higher BC dosages (Fig. 3). The products produced by sucrose synthase through sucrose cleavage are used for energy production, primary metabolite production and complex carbohydrate synthesis, thereby promoting plant growth. The plants overexpressed the SS gene and exhibited improved growth [49]. The sucrose-synthesizing enzyme ensures the demand for sucrose and affects the carbon partitioning to starch, and a higher activity of this enzyme is positively correlated with biomass accumulation in plants [50]. The increased activity of these enzymes corroborates the biomass accumulation in flue-cured tobacco plants, as the activity was highest at the 600 kg/ha BC dosage (Fig. 4). The plants accumulated more biomass until 100 d; therefore, the dry matter content was greater than that at 60 d after transplantation. However, the biomass accumulation in roots was greater than that in stems and leaves.

Although plant growth is affected by BC dosage, dose-dependent studies to elucidate the growth response are relatively scarce. Our results highlight the dose-dependent effect of BC on flue-cured tobacco plants, but the molecular mechanism underlying the negative impact of higher BC dosages is still elusive. Therefore, transcriptome analysis was performed for an effective dosage of 600 kg/ha and a detrimental dosage of 1800 kg/ha to understand the fundamental molecular events underlying these plant responses. DEGs from various comparison groups were analyzed through GO and KEGG pathway analysis (Figures S4 and S5). Finally, DEGs involved in targeted pathways, photosynthesis, carbon metabolism, and sucrose and starch metabolism were further studied in the PCK vs. PA1 and PA1 vs. PA4 comparison groups (Fig. 7). Important transcripts encoding intrinsic proteins such as PsbB, Psb28, PsbO, PsbW, PsbS, and PsbQ were highly up-regulated in PA1 compared with PCK and downregulated in PA4 compared with PA1 (Table S4 and Table S5). These proteins are involved in the function and assembly of photosystem II in plants, and deletion mutants of any of these genes result in defects in the assembly of photosystem II supercomplexes and inefficient photosynthesis [51, 52]. In photosystem I, among four reaction center proteins (G, H, N, O) [53], transcripts related to three of those proteins (PsaG, PsaH and PsaN) was significantly up-regulated in PA1 compared with PCK (Fig. 8) and downregulated in PA4 compared with PA1. Other necessary transcripts related to the cytochrome b6/f complex, the gamma chain of ATP synthase and the photosynthetic electron transport chain were also up-regulated in PCK vs. PA1 compared with PA1 vs. PA4. Our results are in accordance with a recent study on pepper in which BC application also up-regulated genes related to photosynthesis [54]. These insights from a molecular perspective highlight photosynthesis regulation under BC application. According to a meta-analysis, BC application enhanced the photosynthetic rate by 23% on average [55].

Following photosynthesis (energy-producing reactions), carbon from carbon dioxide is fixed through the Calvin cycle in light-independent reactions. Several transcripts encoding key regulatory enzymes, such as ribulose bisphosphate carboxylase, fructose-1,6-bisphosphatase, glyceraldehyde-3-phosphate dehydrogenase, fructose-bisphosphate aldolase, transketolase, sedoheptulose-1,7-bisphosphatase [56] and enzymes (phosphoenolpyruvate carboxylase and malate dehydrogenase) involved in the dicarboxylic acid cycle [57], were up-regulated in PCK vs. PA1 (Figure S6 and Table S6) compared with PA1 vs. PA4 (Table S7). The upregulation of these enzymes indicates the promotion of the carbon fixation pathway and the production of carbohydrate products after BC application, while a higher dosage (PA4) inhibits these pathways. A metabolomic analysis of pepper plants amended with BC further confirmed the accumulation of D-fructose-1,6-biphosphate and ribulose 5-phosphate, which are reactants of Calvin cycle enzymes [54]. Similarly, increased activity of sedoheptulose-1,7-bisphosphatase in transgenic tobacco enhanced photosynthesis and plant growth. The leaf area also increased compared with that of the control plant [58], which indicates that the increase in the activity of this enzyme might be involved in the increase in leaf area after BC application.

At the end of the Calvin cycle, carbon dioxide is efficiently converted to 3-phosphoglyceric acid and glyceraldehyde-3-phosphate, which serve as precursors for sucrose and starch biosynthesis. Starch is temporarily stored in chloroplasts during the day, and different enzymes transform it into sucrose at night to transport it to sink tissue. [59]. Several enzymes involved in starch synthesis and breakdown, such as starch synthase, beta-amylase, 4-alpha-glucanotransferase DPE2, fructokinase, isoamylase, and beta-glucosidase [60], were up-regulated in PCK vs. PA1 (Figure S7 and Table S8) but downregulated in PA1 vs. PA4 (Table S9). When starch is mobilized to sink tissue as sucrose, sucrose is utilized for plant growth and development through various metabolic pathways. Key enzymes for sucrose synthesis, sucrose phosphate synthase, sucrose phosphatase, and sucrose breakdown, acid beta-fructofuranosidase, also known as invertase, were significantly up-regulated in PCK vs. PA1 and downregulated in PA1 vs. PA4. Sucrose is hydrolyzed for energy, metabolic compounds, and complex carbohydrate synthesis [49]. A crucial source-sink balance is essential for optimal plant growth and development. The upregulation of enzymes involved in starch and sucrose metabolism in PA1 (600 kg/ha) highlights that the balance was maintained and that more biomass accumulated in tobacco plants because of improved photosynthesis and carbon assimilation. However, the higher dosage might have disturbed the source‒sink balance, thereby halting growth.

## Conclusion

In conclusion, an optimal dose of BC for flue-cured tobacco is conducive to growth and biomass accumulation. Our study highlights the dose-dependent relationship, where 600 kg/ha promoted growth in flue-cured tobacco plants and a higher dosage inhibited optimal growth. Furthermore, transcriptome analysis provided insight into the underlying molecular mechanisms involved. Important pathways, such as photosynthesis, carbon fixation, and sucrose and starch metabolism, were differentially regulated in plants treated with optimal and high BC dosages compared with those in control plants. The improved growth and biomass accumulation were due to improved photosynthesis and carbon fixation under optimal BC application. The sucrose and starch partitioning in the source-sink balance also played a role in increasing biomass accumulation when BC was applied to tobacco plants. This study provides in-depth knowledge about the molecular mechanisms involved in the BC dose-dependent relationship with tobacco plants and lays a foundation for promoting BC application for growth promotion in plants.

### Electronic supplementary material

Below is the link to the electronic supplementary material.

Supplementary Material 1

## Data Availability

The raw RNA sequencing data have been deposited in the Sequence Read Archive (SRA) in the NCBI public database under BioProject No. PRJNA1018062.

## Declarations

### Statement on guidelines

All experimental studies and experimental materials involved in this research are in full compliance with relevant institutional, national and international guidelines and legislation.
