# Supplementary material for: Transcriptome profiling reveals the impact of various levels of biochar application on the growth of flue-cured tobacco plants
Source: BMC Plant Biol. 2024 Jul 10;24:655. doi: 10.1186/s12870-024-05321-z (PMC11234667; doi:10.1186/s12870-024-05321-z)
Supplement: Supplementary file 1 — Supplementary Material 1 [file 12870_2024_5321_MOESM1_ESM.docx]

**Transcriptome profiling reveals the impact of various levels of biochar application on the growth of flue-cured tobacco**

Yingfen Yang^a,^†, Waqar Ahmed^a,^†, Gang Wang^b^, Chenghu Ye^c^, Shichen Li^a^, Meiwei Zhao^a^, Jinhao Zhang^a^, Junjie Wang^b^, Saleh H. Salmen^d^, Lianzhang Wu^e^, and Zhengxiong Zhao^a^*

^a^Yunnan Agricultural University, Kunming 650201, Yunnan, China

^b^Jiangsu Key Laboratory for Bioresources of Saline Soils, Yancheng Teachers University, Yancheng 224007, China

^c^Yunnan Revert Medical and Biotechnology Co., Ltd., Kunming 65021, Yunnan, China

^d^Department of Botany and Microbiology, College of Science, King Saud University, Riyadh 11451, Saudi Arabia

^e^Nujiang Green Spice Industry Research Institute, Lushui 673200, Yunnan, China

† Authors contributed equally to this work and share first authorship.

***Corresponding authors**

**Name:** Zhengxiong Zhao

**Email:** zhaozx0801@163.com

| **Table S1.** Primers used for RNA-seq validation through qRT-PCR. | | | |
| --- | --- | --- | --- |
| Gene-Id | sequence（5‘-3’） | | product size |
| gene_32 | F | TCCTAGCTTCTTCTCAGATGTGT | 106bp |
|  | R | CGGTGCGGTAAGTGGAGAAT |  |
| gene_15058 | F | TGTTTGTTGATGGTCCGGGT | 166bp |
|  | R | GTGCAAGAGGCATTGCTTACG |  |
| gene_35004 | F | CCAGGTTACTACGATGGAAGGT | 122bp |
|  | R | ATCCAGGCTTGTGGGTAAGC |  |
| gene_11368 | F | AAAGCTCCTGCCAAAAAGGTTG | 119bp |
|  | R | GCAACACGGCCAACAAAGAG |  |
| gene_22686 | F | CCATTTTCAGGCACTGTTGCT | 141bp |
|  | R | CACTTCCTCTTTCTTAATCCCTGG |  |
| gene_71064 | F | ACCTGAGCTCTTCATTCGCC | 105bp |
|  | R | CCCAAATTGTTGACCAAATCTGC |  |
| gene_71328 | F | CCTCTGGTGGATTACCTGGAA | 118bp |
|  | R | TTAACTCTCTGGGCTGCCTC |  |
| gene_18443 | F | TGTAGGTGCAGTTGTAGTTGCTA | 175bp |
|  | R | GTTGTGGGGGAAACCGGC |  |
| gene_39177 | F | AGCAGTGAGGAGAAGTCGCT | 154bp |
|  | R | TCTTGGGAGGAACAGCAACAG |  |
| gene_2398 | F | GCCCCAGCTAGAAACAACCT | 198bp |
|  | R | AGCATTTGCTCCTGAGACGA |  |
| gene_43545 | F | ACAACCCTCTTCAGAGCAAGT | 122bp |
|  | R | TGCTGTCGAACTGAAGTAAGCA |  |
| gene_17565 | F | GTCAAGTGCATGGCTACAAGC | 164bp |
|  | R | CTACCACCCCCTGACCCAG |  |
| gene_30863 | F | GCCCCTCATCTCTTCTTGGATTG | 101bp |
|  | R | CATTCCCAACTTTGATGTTCCCC |  |
| gene_77667 | F | CCATACAACTCTCTTCAGAGCAA | 120bp |
|  | R | CGAACTGAAGTAGGCAAGGGT |  |
| gene_32839 | F | AACCAAGCGCAGATTTGACA | 106bp |
|  | R | AAACTCTCCCCAGTGCCTTT |  |
| Actin | F | AGATGCCTATGTGGGTGAC | 213bp |
|  | R | TGGGTCATTTTCTCTCTGTTG |  |

| **Table S2.** Data obtained from the RNA-seq of 18 samples (3 treatments × 2 timepoints × 3 biological replications per treatment) on the Illumina HiSeq platform. | | | | | | | | | | | | |
| --- | --- | --- | --- | --- | --- | --- | --- | --- | --- | --- | --- | --- |
| **Sample name** | **Sample ID** | **Raw reads (#)** | **Clean reads (#)** | **Mapped reads (#)** | **Mapped reads (%)** | **Unique mapped reads (#)** | **Unique mapped reads (%)** | **Unmapped reads (#)** | **Unmapped reads (%)** | **Q20 (%)** | **Q30 (%)** | **GC (%)** |
| PCK | PCK-1 | 38315022 | 38028136 | 31575850 | 90.89 | 30650839 | 88.22 | 3166518 | 9.11 | 97.51 | 93.13 | 43.82 |
|  | PCK-2 | 37519964 | 37238536 | 32845656 | 92.84 | 31936719 | 90.27 | 2533908 | 7.16 | 97.58 | 93.19 | 43.36 |
|  | PCK-3 | 52502586 | 52021614 | 43730239 | 91.36 | 42413992 | 88.61 | 4133489 | 8.64 | 97.67 | 93.54 | 44.08 |
| PA1 | PA1-1 | 39385078 | 38901070 | 26197959 | 91.24 | 25481097 | 88.75 | 2514263 | 8.76 | 97.37 | 92.9 | 46.51 |
|  | PA1-2 | 47851110 | 47400032 | 31922021 | 93.11 | 30972381 | 90.34 | 2363681 | 6.89 | 97.61 | 93.42 | 46.94 |
|  | PA1-3 | 36274180 | 35861922 | 23680044 | 92.73 | 22998268 | 90.06 | 1857674 | 7.27 | 97.60 | 93.38 | 46.72 |
| PA4 | PA4-1 | 42554630 | 42268344 | 37160647 | 91.93 | 36046031 | 89.17 | 3262061 | 8.07 | 97.58 | 93.16 | 43.34 |
|  | PA4-2 | 40678924 | 40386640 | 35359832 | 92.54 | 34312936 | 89.80 | 2848480 | 7.46 | 97.60 | 93.25 | 43.69 |
|  | PA4-3 | 40055188 | 39697928 | 35491579 | 92.44 | 34473529 | 89.79 | 2901447 | 7.56 | 97.65 | 93.41 | 43.25 |
| MCK | MCK-1 | 43099112 | 42820582 | 39240223 | 92.38 | 38184345 | 89.89 | 3238729 | 7.62 | 97.61 | 93.23 | 42.84 |
|  | MCK-2 | 46780688 | 46436214 | 42827206 | 93.15 | 41642522 | 90.57 | 3149612 | 6.85 | 97.68 | 93.44 | 42.86 |
|  | MCK-3 | 39676772 | 39385446 | 36263150 | 93.32 | 35288123 | 90.81 | 2594558 | 6.68 | 97.66 | 93.35 | 42.83 |
| MA1 | MA1-1 | 43226266 | 42950556 | 39137147 | 92.70 | 38115653 | 90.28 | 3080845 | 7.30 | 97.60 | 93.14 | 42.97 |
|  | MA1-2 | 50479642 | 50171496 | 46302900 | 93.23 | 45089572 | 90.79 | 3362756 | 6.77 | 98.05 | 94.11 | 42.82 |
|  | MA1-3 | 40388176 | 40142708 | 36916691 | 93.39 | 35872346 | 90.74 | 2614447 | 6.61 | 97.87 | 93.67 | 43.01 |
| MA4 | MA4-1 | 42882954 | 42589476 | 38957800 | 92.64 | 37841258 | 89.99 | 3093902 | 7.36 | 97.68 | 93.42 | 43.05 |
|  | MA4-2 | 48965502 | 48678576 | 45025396 | 93.59 | 43760743 | 90.96 | 3085500 | 6.41 | 98.04 | 94.11 | 42.99 |
|  | MA4-3 | 43243936 | 42983984 | 39750400 | 93.13 | 38671083 | 90.60 | 2934558 | 6.87 | 97.87 | 93.67 | 42.86 |
| Total |  | 773879730 | 767963260 | 662384740 |  | 643751437 |  | 52736428 |  |  |  |  |
| Average |  | 42993318 | 42664626 | 36799152 | 92.59 | 35763969 | 89.98 | 2929802 | 7.41 | 97.68 | 93.42 | 43.77 |
| Raw reads: are the reads obtained through sequencing of 18 samples on Illumina HiSeq platform. Clean reads: are the reads obtained from removing low-quality reads and adaptor sequences from raw reads. Mapped reads: the number of clean reads aligned to the reference genome and the percentage of clean reads. Unique mapped reads: the number of clean reads aligned to the reference genome in the unique position and the percentage of clean reads. Unmapped reads: the number of unaligned reads and their percentage. Q20% and Q30%; the percentage of bases with a clean data quality value greater than or equal to 20 and 30, respectively. GC (%): clean data GC contents percentage. Here; PCK, PA1, and PA4 represents the samples collected under 0, 600, and 1800 kg/ha application rate of biochar, respectively after 60 days of post-transplantation. MCK, MA1, and MA4 represents the samples collected under 0, 600, and 1800 kg/ha application rate of biochar, respectively after 100 days of post-transplantation. | | | | | | | | | | | | |

| **Table S3.** Differential expressed genes in groupwise comparison. | | | |
| --- | --- | --- | --- |
| **Groups** | **Up-regulated** | **Down-regulated** | **Total** |
| PCK-vs-PA1 | 2417 | 4144 | 6561 |
| PCK-vs-PA4 | 713 | 790 | 1503 |
| PA1-vs-PA4 | 6544 | 5504 | 12048 |
| PCK-vs-MCK | 4639 | 3433 | 8072 |
| PA1-vs-MA1 | 8805 | 4763 | 13568 |
| PA4-vs-MA4 | 7604 | 3742 | 11346 |
| MCK-vs-MA1 | 53 | 40 | 93 |
| MCK-vs-MA4 | 110 | 136 | 246 |
| MA1-vs-MA4 | 43 | 53 | 96 |
| Total | 30928 | 22605 | 53533 |
| Here; PCK, PA1, and PA4 represents the samples collected under 0, 600, and 1800 kg/ha application rate of biochar, respectively after 60 days of post-transplantation. MCK, MA1, and MA4 represents the samples collected under 0, 600, and 1800 kg/ha application rate of biochar, respectively after 100 days of post-transplantation. | | | |

| **Table S4. Gene regulation in photosynthesis pathway from groupwise comparison between PCK vs PA1.** | | | | | | |
| --- | --- | --- | --- | --- | --- | --- |
| **Gene-Id** | **log2(Fc)** | **P-Value** | **FDR** | **Status** | **Symbol** | **Description** |
| gene_11368 | 1.713921377 | 6.03E-13 | 9.82E-11 | Up-regulated | PSBS | photosystem II 22 kDa protein |
| gene_29908 | 1.731066195 | 2.81E-11 | 3.26E-09 | Up-regulated | PSBW | photosystem II reaction center W protein |
| gene_59741 | 1.32417623 | 2.57E-10 | 2.42E-08 | Up-regulated | ATPC | ATP synthase gamma chain |
| gene_84556 | 1.554701086 | 2.97E-10 | 2.73E-08 | Up-regulated | PSB28 | photosystem II reaction center Psb28 protein-like |
| gene_32839 | 1.423367208 | 7.57E-10 | 6.37E-08 | Up-regulated | PSAF | photosystem I reaction center subunit III |
| gene_21116 | 1.530652433 | 3.17E-09 | 2.35E-07 | Up-regulated | PNSL2 | photosynthetic NDH subunit of lumenal location 2 |
| gene_43545 | 1.586460404 | 3.42E-09 | 2.51E-07 | Up-regulated | PSAH | photosystem I reaction center subunit VI-1 |
| gene_74439 | 1.424928347 | 3.82E-09 | 2.75E-07 | Up-regulated | PNSL3 | photosynthetic NDH subunit of lumenal location 3 |
| gene_677 | 1.297975872 | 7.89E-09 | 5.05E-07 | Up-regulated | PETH | ferredoxin--NADP reductase, leaf-type isozyme |
| gene_46518 | 1.753607292 | 2.64E-08 | 1.47E-06 | Up-regulated | PSAF | photosystem I reaction center subunit III |
| gene_73192 | 1.265160806 | 7.46E-08 | 3.63E-06 | Up-regulated | PNSL3 | photosynthetic NDH subunit of lumenal location 3 |
| gene_35277 | 1.267827893 | 9.13E-08 | 4.32E-06 | Up-regulated | PSBO | oxygen-evolving enhancer protein 1 |
| gene_71328 | 1.260162076 | 1.88E-07 | 8.08E-06 | Up-regulated | PSBQ2 | oxygen-evolving enhancer protein 3-2 |
| gene_2398 | 1.399921314 | 2.53E-07 | 1.05E-05 | Up-regulated | PSBO | oxygen-evolving enhancer protein 1 |
| gene_36597 | 1.176795479 | 2.93E-07 | 1.18E-05 | Up-regulated | PSBO | oxygen-evolving enhancer protein 1 |
| gene_17565 | 1.105652208 | 4.03E-07 | 1.53E-05 | Up-regulated | petC2 | cytochrome b6-f complex iron-sulfur subunit 2 |
| gene_72177 | 1.232510755 | 8.18E-07 | 2.79E-05 | Up-regulated | PSBO | oxygen-evolving enhancer protein 1 |
| gene_328 | 1.302569624 | 9.45E-07 | 3.16E-05 | Up-regulated | PSAK | photosystem I reaction center subunit psaK |
| gene_66199 | 1.215192229 | 1.43E-06 | 4.51E-05 | Up-regulated | PETH | ferredoxin--NADP reductase, leaf-type isozyme |
| gene_78629 | 1.693101454 | 3.07E-06 | 8.66E-05 | Up-regulated | PSBO | oxygen-evolving enhancer protein 1 |
| gene_65015 | 1.277548956 | 3.26E-06 | 9.12E-05 | Up-regulated | PSAK | photosystem I reaction center subunit psaK |
| gene_30863 | 1.299126689 | 3.38E-06 | 9.40E-05 | Up-regulated | PSBW | photosystem II reaction center W protein |
| gene_17490 | 1.42420462 | 4.05E-06 | 0.000109287 | Up-regulated | PSB28 | photosystem II reaction center Psb28 protein |
| gene_34967 | 1.037817767 | 9.79E-06 | 0.000232073 | Up-regulated | PSBW | photosystem II reaction center W protein |
| gene_59136 | 1.240843856 | 1.09E-05 | 0.000254053 | Up-regulated | PSAH2 | photosystem I reaction center subunit VI-2 |
| gene_80940 | 1.187964733 | 1.48E-05 | 0.00032566 | Up-regulated | PSAK | photosystem I reaction center subunit psaK |
| gene_22727 | 1.006915589 | 1.66E-05 | 0.000358662 | Up-regulated | PSAN | photosystem I reaction center subunit N |
| gene_79012 | 1.131643393 | 2.07E-05 | 0.0004319 | Up-regulated | PSAH2 | photosystem I reaction center subunit VI-2 |
| gene_18443 | 1.041458914 | 4.63E-05 | 0.000831265 | Up-regulated | PETE | plastocyanin B'/B'' |
| gene_77667 | 1.009249588 | 5.71E-05 | 0.000990306 | Up-regulated | PSAH | photosystem I reaction center subunit VI-1 |
| gene_74250 | 1.06754159 | 6.16E-05 | 0.001049342 | Up-regulated | PSAG | photosystem I reaction center subunit V |
| gene_67287 | 1.229611673 | 0.000118135 | 0.001801055 | Up-regulated | PSAK | photosystem I reaction center subunit psaK |
| MSTRG.52080 | 1.362557576 | 0.000183351 | 0.002588945 | Up-regulated | PSAL | photosystem I reaction center subunit XI |
| gene_14491 | 1.383087869 | 0.000204918 | 0.002842359 | Up-regulated | PSBW | photosystem II reaction center W protein |
| gene_78139 | 1.047024331 | 0.00021162 | 0.002913079 | Up-regulated | PSAH1 | photosystem I reaction center subunit VI-1 |
| gene_32307 | 1.185447513 | 0.002028244 | 0.017609433 | Up-regulated | PNSL2 | psbQ-like protein 1 |
| MSTRG.51289 | 3.916721605 | 0.007786659 | 0.049493975 | Up-regulated | psbB | photosystem II 47 kDa protein |

| **Table S5. Genes regulated in photosynthesis pathway from groupwise comparison between PA1 vs PA4.** | | | | | | |
| --- | --- | --- | --- | --- | --- | --- |
| **Gene- Id** | **log2(Fc)** | **P-Value** | **FDR** | **Status** | **Symbol** | **Description** |
| gene_11368 | -1.504205843 | 6.63E-11 | 1.21E-09 | Down-regulated | PSBS | photosystem II 22 kDa protein |
| MSTRG.52080 | -1.882013683 | 2.44E-17 | 9.03E-16 | Down-regulated | PSAL | photosystem I reaction center subunit XI |
| gene_78629 | -3.244185349 | 4.73E-14 | 1.27E-12 | Down-regulated | PSBO | oxygen-evolving enhancer protein 1 |
| gene_71328 | -1.439442643 | 1.29E-13 | 3.28E-12 | Down-regulated | PSBQ2 | oxygen-evolving enhancer protein 3-2 |
| gene_32839 | -1.499516896 | 3.53E-13 | 8.57E-12 | Down-regulated | PSAF | photosystem I reaction center subunit III |
| gene_59136 | -1.419978889 | 3.77E-13 | 9.15E-12 | Down-regulated | PSAH2 | photosystem I reaction center subunit VI-2 |
| gene_74250 | -1.534275202 | 8.31E-13 | 1.93E-11 | Down-regulated | PSAG | photosystem I reaction center subunit V |
| gene_59741 | -1.493794167 | 1.94E-12 | 4.32E-11 | Down-regulated | ATPC | ATP synthase gamma chain |
| gene_36597 | -1.386394383 | 1.13E-11 | 2.31E-10 | Down-regulated | PSBO | oxygen-evolving enhancer protein 1 |
| gene_46518 | -2.112599235 | 4.59E-27 | 3.47E-25 | Down-regulated | PSAF | photosystem I reaction center subunit III |
| gene_21116 | -1.762853315 | 1.95E-10 | 3.37E-09 | Down-regulated | PNSL2 | photosynthetic NDH subunit of lumenal location 2 |
| gene_29908 | -1.657972147 | 3.16E-10 | 5.29E-09 | Down-regulated | PSBW | photosystem II reaction center W protein |
| gene_35277 | -1.374984002 | 4.75E-10 | 7.76E-09 | Down-regulated | PSBO | oxygen-evolving enhancer protein 1 |
| gene_17565 | -1.188361238 | 4.83E-10 | 7.87E-09 | Down-regulated | petC2 | cytochrome b6-f complex iron-sulfur subunit 2 |
| gene_74439 | -1.443723494 | 5.18E-10 | 8.42E-09 | Down-regulated | PNSL3 | photosynthetic NDH subunit of lumenal location 3 |
| gene_2398 | -1.184418869 | 6.46E-10 | 1.03E-08 | Down-regulated | PSBO | oxygen-evolving enhancer protein 1 |
| gene_43545 | -1.146000342 | 6.86E-09 | 9.57E-08 | Down-regulated | PSAH | photosystem I reaction center subunit VI-1 |
| gene_677 | -1.207262186 | 7.45E-09 | 1.03E-07 | Down-regulated | PETH | ferredoxin--NADP reductase, leaf-type isozyme |
| gene_30863 | -1.347259185 | 7.60E-09 | 1.05E-07 | Down-regulated | PSBW | photosystem II reaction center W protein |
| gene_328 | -1.327674587 | 3.67E-08 | 4.60E-07 | Down-regulated | PSAK | photosystem I reaction center subunit psaK |
| gene_73192 | -1.192829788 | 6.53E-08 | 7.87E-07 | Down-regulated | PNSL3 | photosynthetic NDH subunit of lumenal location 3 |
| gene_65015 | -1.183338895 | 7.55E-08 | 8.99E-07 | Down-regulated | PSAK | photosystem I reaction center subunit psaK |
| gene_72177 | -1.061532067 | 1.07E-07 | 1.24E-06 | Down-regulated | PSBO | oxygen-evolving enhancer protein 1 |
| gene_77667 | -1.013488302 | 2.06E-07 | 2.28E-06 | Down-regulated | PSAH | photosystem I reaction center subunit VI-1 |
| gene_34967 | -1.191995479 | 4.07E-07 | 4.27E-06 | Down-regulated | PSBW | photosystem II reaction center W protein |
| gene_18443 | -1.326591823 | 7.39E-07 | 7.42E-06 | Down-regulated | PETE | plastocyanin B'/B'' |
| gene_80940 | -1.1863496 | 1.54E-06 | 1.46E-05 | Down-regulated | PSAK | photosystem I reaction center subunit psaK |
| gene_22727 | -1.107635667 | 3.61E-06 | 3.17E-05 | Down-regulated | PSAN | photosystem I reaction center subunit N |
| gene_79012 | -1.004145263 | 7.64E-06 | 6.30E-05 | Down-regulated | PSAH2 | photosystem I reaction center subunit VI-2 |
| gene_66199 | -1.062978507 | 1.33E-05 | 0.000104583 | Down-regulated | PETH | ferredoxin--NADP reductase, leaf-type isozyme |
| gene_84556 | -1.240177279 | 1.56E-05 | 0.000120195 | Down-regulated | PSB28 | photosystem II reaction center Psb28 protein-like |
| gene_32307 | -1.345339481 | 5.17E-05 | 0.000356381 | Down-regulated | PNSL2 | psbQ-like protein 1, chloroplastic isoform X2 |
| gene_78139 | -1.044501735 | 7.95E-05 | 0.000524441 | Down-regulated | PSAH1 | photosystem I reaction center subunit VI-1 |
| gene_67287 | -1.206164012 | 0.000121813 | 0.000766107 | Down-regulated | PSAK | photosystem I reaction center subunit psaK |
| gene_17490 | -1.295546603 | 0.000562017 | 0.002958243 | Down-regulated | PSB28 | photosystem II reaction center Psb28 protein |
| gene_14491 | -1.560253514 | 0.000787116 | 0.003967361 | Down-regulated | PSBW | photosystem II reaction center W protein |
| MSTRG.51289 | -3.150461704 | 0.015119163 | 0.049624647 | Down-regulated | psbB | photosystem II 47 kDa protein |

| **Table S6. Genes regulated in Carbon fixation in photosynthetic organism pathway from groupwise comparison between PCK vs PA1.** | | | | | | |
| --- | --- | --- | --- | --- | --- | --- |
| **Gene-Id** | **log2(Fc)** | **P-Value** | **FDR** | **Status** | **Symbol** | **Description** |
| gene_27987 | 2.642925338 | 4.01E-28 | 4.98E-25 | Up-regulated | FBPban1 | fructose-1,6-bisphosphatase |
| gene_15058 | 2.864252536 | 1.41E-27 | 1.59E-24 | Up-regulated | GAPB | glyceraldehyde-3-phosphate dehydrogenase B |
| gene_70172 | 2.49413879 | 2.90E-27 | 3.18E-24 | Up-regulated | FBPban1 | fructose-1,6-bisphosphatase, cytosolic |
| MSTRG.51903 | 2.369433895 | 7.21E-22 | 4.36E-19 | Up-regulated | RBCS | ribulose bisphosphate carboxylase small chain S41 |
| gene_68511 | 2.121626097 | 2.99E-17 | 1.06E-14 | Up-regulated | GAPB | glyceraldehyde-3-phosphate dehydrogenase B |
| gene_71228 | 1.925254515 | 8.36E-17 | 2.68E-14 | Up-regulated | TKL-1 | Transketolase |
| gene_79620 | 2.319715372 | 4.66E-16 | 1.34E-13 | Up-regulated | OsI_10887 | fructose-1,6-bisphosphatase |
| gene_3051 | 2.256040159 | 6.44E-16 | 1.83E-13 | Up-regulated | At1g32060 | Phosphoribulokinase |
| gene_14024 | 1.675046552 | 2.59E-15 | 6.59E-13 | Up-regulated | RBCS | ribulose bisphosphate carboxylase small chain |
| gene_76758 | 2.059314729 | 7.12E-15 | 1.67E-12 | Up-regulated | GGAT2 | glutamate-glyoxylate aminotransferase 2 |
| gene_30765 | 1.748733477 | 8.45E-15 | 1.96E-12 | Up-regulated | GAPA | glyceraldehyde-3-phosphate dehydrogenase A |
| gene_47109 | 1.786734167 | 6.71E-14 | 1.28E-11 | Up-regulated | At3g55800 | sedoheptulose-1,7-bisphosphatase |
| gene_10489 | 1.823879367 | 6.59E-13 | 1.06E-10 | Up-regulated | TKL-1 | Transketolase |
| gene_42407 | 1.487448738 | 3.38E-11 | 3.83E-09 | Up-regulated | At3g55800 | sedoheptulose-1,7-bisphosphatase |
| gene_57160 | 1.670470477 | 9.45E-11 | 9.68E-09 | Up-regulated | FBA2 | fructose-bisphosphate aldolase 1 |
| gene_66218 | 1.565800497 | 4.40E-10 | 3.90E-08 | Up-regulated | GAPA | glyceraldehyde-3-phosphate dehydrogenase A |
| gene_61700 | 1.64789495 | 1.47E-09 | 1.17E-07 | Up-regulated | PPC1 | phosphoenolpyruvate carboxylase |
| gene_65990 | 1.368460216 | 2.14E-09 | 1.66E-07 | Up-regulated | OsI_10887 | fructose-1,6-bisphosphatase |
| gene_57322 | 1.868656429 | 4.18E-09 | 2.97E-07 | Up-regulated | FBA2 | fructose-bisphosphate aldolase 1 |
| gene_21580 | 1.532594985 | 4.65E-09 | 3.23E-07 | Up-regulated | GGAT2 | glutamate--glyoxylate aminotransferase 2 |
| gene_44833 | 1.256698671 | 1.14E-08 | 6.95E-07 | Up-regulated | RBCS | ribulose bisphosphate carboxylase small chain |
| gene_61363 | 1.297971998 | 2.00E-08 | 1.15E-06 | Up-regulated | RBCS | ribulose bisphosphate carboxylase small chain S41 |
| gene_59631 | 1.225495831 | 4.33E-08 | 2.25E-06 | Up-regulated | At1g32060 | phosphoribulokinase |
| gene_82004 | 1.745943382 | 4.95E-08 | 2.53E-06 | Up-regulated | FBPban1 | fructose-1,6-bisphosphatase |
| gene_62167 | 1.264090275 | 8.46E-08 | 4.05E-06 | Up-regulated | FBA2 | fructose-bisphosphate aldolase 1 |
| gene_23845 | 1.082679206 | 2.74E-07 | 1.12E-05 | Up-regulated | At3g55800 | sedoheptulose-1,7-bisphosphatase |
| gene_44694 | 1.424781818 | 5.03E-07 | 1.84E-05 | Up-regulated | FBPban1 | fructose-1,6-bisphosphatase |
| gene_38021 | 1.608103374 | 6.23E-07 | 2.21E-05 | Up-regulated | FBA2 | fructose-bisphosphate aldolase 1 |
| gene_33501 | 1.137470032 | 8.99E-07 | 3.02E-05 | Up-regulated | MDHG | malate dehydrogenase |
| gene_60848 | 1.627259607 | 1.10E-06 | 3.59E-05 | Up-regulated | FBA2 | fructose-bisphosphate aldolase 1 |
| gene_48526 | 1.052718599 | 2.23E-06 | 6.57E-05 | Up-regulated | At1g32060 | phosphoribulokinase |
| gene_73919 | 1.54628312 | 3.92E-06 | 0.000106395 | Up-regulated | FBA2 | fructose-bisphosphate aldolase 1 |
| gene_76999 | 1.070786488 | 4.66E-06 | 0.000123257 | Up-regulated | RPI4 | probable ribose-5-phosphate isomerase 4 |
| gene_43822 | 1.143130565 | 5.34E-06 | 0.000138819 | Up-regulated | PPC4 | phosphoenolpyruvate carboxylase 4-like |
| gene_71554 | 1.315241243 | 8.68E-06 | 0.000209105 | Up-regulated | MDHG | malate dehydrogenase |
| gene_48874 | 1.030410015 | 2.31E-05 | 0.000471923 | Up-regulated | At3g55800 | sedoheptulose-1,7-bisphosphatase |
| gene_65991 | 1.747678725 | 3.61E-05 | 0.000683733 | Up-regulated | FBP | fructose-1,6-bisphosphatase |
| MSTRG.34402 | 1.138856067 | 0.000626826 | 0.006977993 | Up-regulated | -- | hypothetical protein |
| gene_67033 | 1.286621226 | 0.007171145 | 0.046676703 | Up-regulated | FBP | fructose-1,6-bisphosphatase |

| **Table S7. Genes regulated in Carbon fixation in photosynthetic organism pathway in groupwise comparison between PA1 vs PA4.** | | | | | | |
| --- | --- | --- | --- | --- | --- | --- |
| **Gene-Id** | **log2(Fc)** | **P-Value** | **FDR** | **Status** | **Symbol** | **Description** |
| gene_27987 | -1.989951382 | 1.94E-26 | 1.39E-24 | Down-regulated | FBPban1 | fructose-1,6-bisphosphatase |
| gene_15058 | -2.541652012 | 6.30E-37 | 8.37E-35 | Down-regulated | GAPB | glyceraldehyde-3-phosphate dehydrogenase B |
| gene_61700 | -2.69973746 | 9.46E-32 | 9.29E-30 | Down-regulated | PPC1 | phosphoenolpyruvate carboxylase |
| gene_3051 | -2.885975543 | 2.83E-31 | 2.68E-29 | Down-regulated | At1g32060 | phosphoribulokinase |
| gene_65990 | -2.382917406 | 4.49E-25 | 2.94E-23 | Down-regulated | OsI_10887 | fructose-1,6-bisphosphatase |
| gene_68511 | -2.179898561 | 8.67E-24 | 5.24E-22 | Down-regulated | GAPB | glyceraldehyde-3-phosphate dehydrogenase B |
| gene_79620 | -2.193084514 | 2.94E-21 | 1.49E-19 | Down-regulated | OsI_10887 | fructose-1,6-bisphosphatase |
| gene_70172 | -2.125336095 | 5.77E-20 | 2.68E-18 | Down-regulated | FBPban1 | fructose-1,6-bisphosphatase |
| gene_71228 | -1.823227155 | 8.72E-18 | 3.35E-16 | Down-regulated | TKL-1 | transketolase |
| gene_48874 | -1.52908632 | 8.90E-17 | 3.12E-15 | Down-regulated | At3g55800 | sedoheptulose-1,7-bisphosphatase |
| gene_47109 | -1.761169635 | 1.62E-15 | 5.06E-14 | Down-regulated | At3g55800 | sedoheptulose-1,7-bisphosphatase |
| gene_43822 | -1.58819206 | 2.06E-15 | 6.38E-14 | Down-regulated | PPC4 | phosphoenolpyruvate carboxylase 4-like |
| gene_10489 | -1.800136206 | 2.15E-15 | 6.63E-14 | Down-regulated | TKL-1 | transketolase |
| gene_76999 | -1.792059975 | 4.96E-15 | 1.47E-13 | Down-regulated | RPI4 | probable ribose-5-phosphate isomerase 4 |
| gene_76758 | -1.845614924 | 5.16E-15 | 1.52E-13 | Down-regulated | GGAT2 | glutamate--glyoxylate aminotransferase 2 |
| gene_14024 | -1.745085423 | 1.26E-14 | 3.60E-13 | Down-regulated | RBCS | ribulose bisphosphate carboxylase small chain |
| gene_30765 | -1.531204055 | 2.33E-14 | 6.47E-13 | Down-regulated | GAPA | glyceraldehyde-3-phosphate dehydrogenase A |
| gene_57160 | -1.774504106 | 2.72E-13 | 6.67E-12 | Down-regulated | FBA2 | fructose-bisphosphate aldolase 1 |
| gene_62167 | -1.417535779 | 9.68E-13 | 2.23E-11 | Down-regulated | FBA2 | fructose-bisphosphate aldolase 1 |
| gene_42407 | -1.507309771 | 1.96E-12 | 4.36E-11 | Down-regulated | At3g55800 | sedoheptulose-1,7-bisphosphatase |
| gene_82004 | -1.405547132 | 5.92E-12 | 1.25E-10 | Down-regulated | FBPban1 | fructose-1,6-bisphosphatase |
| gene_71554 | -1.399445411 | 2.06E-11 | 4.04E-10 | Down-regulated | MDHG | malate dehydrogenase |
| gene_66218 | -1.304806595 | 5.95E-11 | 1.09E-09 | Down-regulated | GAPA | glyceraldehyde-3-phosphate dehydrogenase A |
| gene_23845 | -1.170071316 | 1.44E-10 | 2.53E-09 | Down-regulated | At3g55800 | sedoheptulose-1,7-bisphosphatase |
| gene_59631 | -1.189515147 | 1.60E-09 | 2.44E-08 | Down-regulated | At1g32060 | phosphoribulokinase |
| gene_48526 | -1.128061198 | 1.46E-08 | 1.94E-07 | Down-regulated | At1g32060 | phosphoribulokinase |
| gene_65991 | -2.414444652 | 3.25E-08 | 4.11E-07 | Down-regulated | FBP | fructose-1,6-bisphosphatase |
| gene_21580 | -1.247562705 | 4.36E-08 | 5.41E-07 | Down-regulated | GGAT2 | glutamate--glyoxylate aminotransferase 2 |
| gene_44833 | -1.1531445 | 1.04E-07 | 1.21E-06 | Down-regulated | RBCS | ribulose bisphosphate carboxylase small chain |
| gene_33501 | -1.185414466 | 1.56E-07 | 1.76E-06 | Down-regulated | MDHG | malate dehydrogenase |
| gene_38021 | -1.632055001 | 1.72E-07 | 1.93E-06 | Down-regulated | FBA2 | fructose-bisphosphate aldolase 1 |
| gene_61363 | -1.234237096 | 2.77E-07 | 3.00E-06 | Down-regulated | RBCS | ribulose bisphosphate carboxylase small chain S41 |
| gene_57322 | -1.443573421 | 8.13E-07 | 8.11E-06 | Down-regulated | FBA2 | fructose-bisphosphate aldolase 1 |
| gene_67033 | -2.524955191 | 6.62E-06 | 5.53E-05 | Down-regulated | FBP | fructose-1,6-bisphosphatase |
| gene_60848 | -1.511866529 | 2.62E-05 | 0.000192494 | Down-regulated | FBA2 | fructose-bisphosphate aldolase 1 |
| gene_44694 | -1.059478295 | 8.14E-05 | 0.000535588 | Down-regulated | FBPban1 | fructose-1,6-bisphosphatase |
| MSTRG.34402 | -1.666574616 | 0.000401056 | 0.002194962 | Down-regulated | -- | hypothetical protein LSAT_6X94260 |
| gene_73919 | -1.287992448 | 0.001159188 | 0.005575239 | Down-regulated | FBA2 | fructose-bisphosphate aldolase 1 |
| MSTRG.51903 | -1.113637986 | 0.005247867 | 0.020483962 | Down-regulated | RBCS | ribulose bisphosphate carboxylase small chain S41 |

| **Table S8. Regulation of DEGs in starch and sucrose metabolic pathway in groupwise comparison between PCK vs PA1.** | | | | | | |
| --- | --- | --- | --- | --- | --- | --- |
| **Gene-Id** | **log2(Fc)** | **P-Value** | **FDR** | **Status** | **Symbol** | **Description** |
| gene_10726 | 2.223569883 | 2.73E-20 | 1.35E-17 | Up-regulated | BAM3 | beta-amylase 3 |
| gene_64744 | 1.938230305 | 6.53E-10 | 5.57E-08 | Up-regulated | SPS4 | probable sucrose-phosphate synthase 4 |
| gene_79812 | 2.011274258 | 1.08E-08 | 6.68E-07 | Up-regulated | BAM3 | beta-amylase 3 |
| gene_52618 | 1.290214619 | 1.43E-07 | 6.35E-06 | Up-regulated | Os10g0521000 | probable trehalase |
| gene_53591 | 1.308282657 | 4.93E-07 | 1.81E-05 | Up-regulated | SPP2 | sucrose-phosphatase 2 |
| gene_6652 | 1.367445011 | 1.33E-06 | 4.25E-05 | Up-regulated | BMY1 | beta-amylase-like |
| gene_59221 | 1.56878862 | 1.94E-06 | 5.85E-05 | Up-regulated | SPS2 | probable sucrose-phosphate synthase 2 isoform X1 |
| gene_18639 | 1.264072087 | 2.94E-06 | 8.32E-05 | Up-regulated | COP10 | constitutive photomorphogenesis protein 10 |
| gene_25774 | 1.169106742 | 4.09E-06 | 0.000110142 | Up-regulated | SS4 | probable starch synthase 4 |
| gene_59986 | 1.636639845 | 1.35E-05 | 0.000302017 | Up-regulated | BAM3 | beta-amylase 3 |
| gene_34222 | 1.358798487 | 1.86E-05 | 0.000394032 | Up-regulated | BGLU40 | beta-glucosidase 40-like |
| gene_27336 | 1.134912728 | 1.96E-05 | 0.000411346 | Up-regulated | DPE2 | 4-alpha-glucanotransferase DPE2-like isoform X1 |
| gene_77157 | 1.180897938 | 9.11E-05 | 0.001448268 | Up-regulated | FRK2 | fructokinase-2 isoform X1 |
| gene_49249 | 2.089161395 | 0.000119673 | 0.00182038 | Up-regulated | BGLU40 | beta-glucosidase 40-like |
| gene_79577 | 1.554729558 | 0.000492348 | 0.005739929 | Up-regulated | BGLU44 | beta-glucosidase 44-like |
| gene_59621 | 1.341982372 | 0.000589883 | 0.006644063 | Up-regulated | TIV1 | acid beta-fructofuranosidase AIV-18-like |
| gene_2797 | 1.136326523 | 0.000746343 | 0.008035373 | Up-regulated | ISA1 | isoamylase 1 |
| gene_7577 | 1.130144905 | 0.001127282 | 0.0111561 | Up-regulated | BMY1 | beta-amylase-like |
| gene_15857 | 1.153378693 | 0.001290548 | 0.012387586 | Up-regulated | ISA1 | isoamylase 1 |

| **Table S9. DEGs from starch and sucrose metabolic pathway from groupwise comparison between PA1 vs PA4.** | | | | | | |
| --- | --- | --- | --- | --- | --- | --- |
| **Gene-Id** | **log2(Fc)** | **P-Value** | **FDR** | **Status** | **Symbol** | **Description** |
| gene_10726 | -3.116735919 | 8.77E-35 | 1.04E-32 | Down-regulated | BAM3 | beta-amylase 3 |
| gene_59986 | -4.005368809 | 3.36E-33 | 3.61E-31 | Down-regulated | BAM3 | beta-amylase 3 |
| gene_15857 | -2.730767974 | 2.64E-26 | 1.87E-24 | Down-regulated | ISA1 | isoamylase 1 |
| gene_79812 | -2.657760335 | 1.31E-19 | 5.87E-18 | Down-regulated | BAM3 | beta-amylase 3 |
| gene_25774 | -1.854524712 | 2.21E-19 | 9.68E-18 | Down-regulated | SS4 | probable starch synthase 4 |
| gene_59621 | -2.021538238 | 2.05E-18 | 8.33E-17 | Down-regulated | TIV1 | acid beta-fructofuranosidase AIV-18-like |
| gene_52618 | -1.820152419 | 1.07E-15 | 3.40E-14 | Down-regulated | Os10g0521000 | probable trehalase |
| gene_2797 | -2.025454994 | 1.50E-13 | 3.78E-12 | Down-regulated | ISA1 | isoamylase 1 |
| gene_79577 | -3.221320821 | 1.99E-12 | 4.42E-11 | Down-regulated | BGLU44 | beta-glucosidase 44-like |
| gene_18639 | -1.684790993 | 4.75E-11 | 8.86E-10 | Down-regulated | COP10 | constitutive photomorphogenesis protein 10 |
| gene_53591 | -1.287387952 | 4.61E-07 | 4.79E-06 | Down-regulated | SPP2 | sucrose-phosphatase 2 |
| gene_49249 | -2.295908636 | 1.82E-06 | 1.70E-05 | Down-regulated | BGLU40 | beta-glucosidase 40-like |
| gene_34222 | -1.50120081 | 3.87E-06 | 3.38E-05 | Down-regulated | BGLU40 | beta-glucosidase 40-like |
| gene_77157 | -1.201574291 | 1.03E-05 | 8.26E-05 | Down-regulated | FRK2 | fructokinase-2 isoform X1 |
| gene_59221 | -1.325864139 | 1.12E-05 | 8.88E-05 | Down-regulated | SPS2 | probable sucrose-phosphate synthase 2 isoform X1 |
| gene_6652 | -1.172105347 | 2.74E-05 | 2.01E-04 | Down-regulated | BMY1 | beta-amylase-like |
| gene_27336 | -1.065738541 | 6.22E-05 | 4.20E-04 | Down-regulated | DPE2 | 4-alpha-glucanotransferase DPE2-like isoform X1 |
| gene_64744 | -1.445072025 | 0.000354223 | 1.97E-03 | Down-regulated | SPS4 | probable sucrose-phosphate synthase 4 |
| gene_7577 | -1.318485684 | 0.000399961 | 2.19E-03 | Down-regulated | BMY1 | beta-amylase-like |


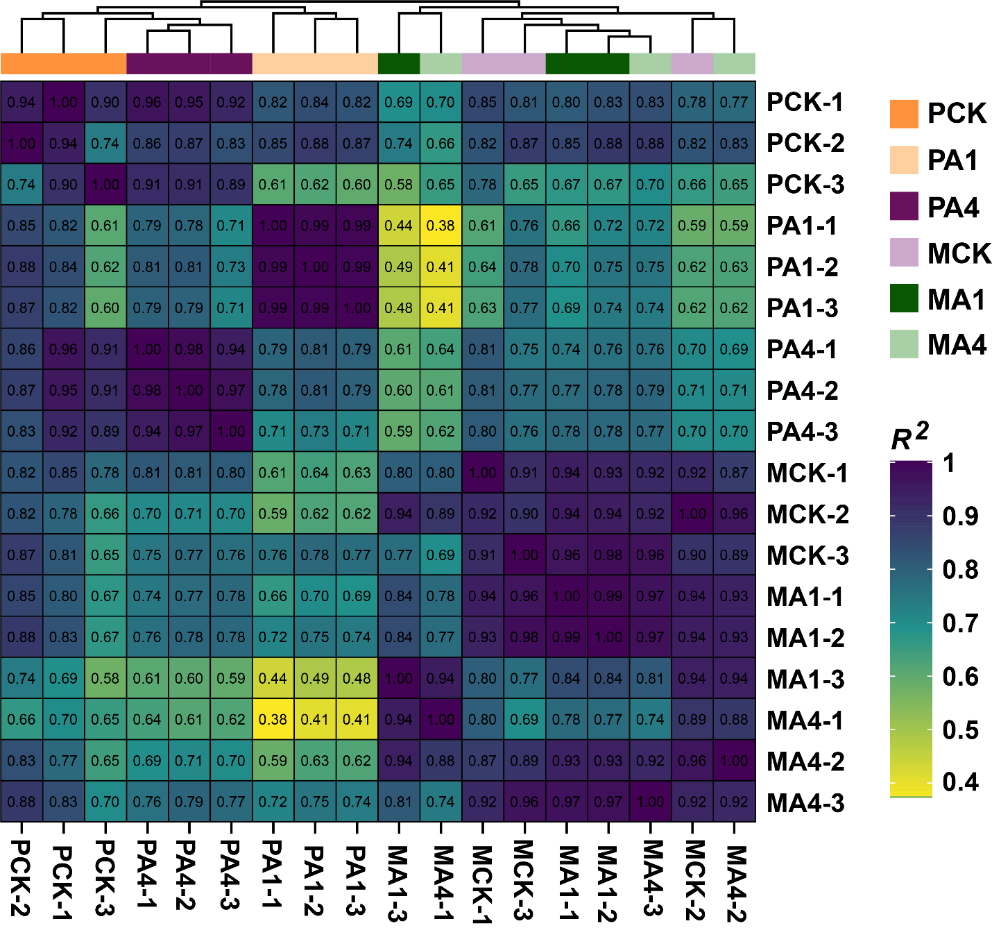


**Figure S1.** Pearson correlation between samples of the treatment groups. The values exhibit the correlation coefficient between two samples where the value of 1 is the highest correlation.

**
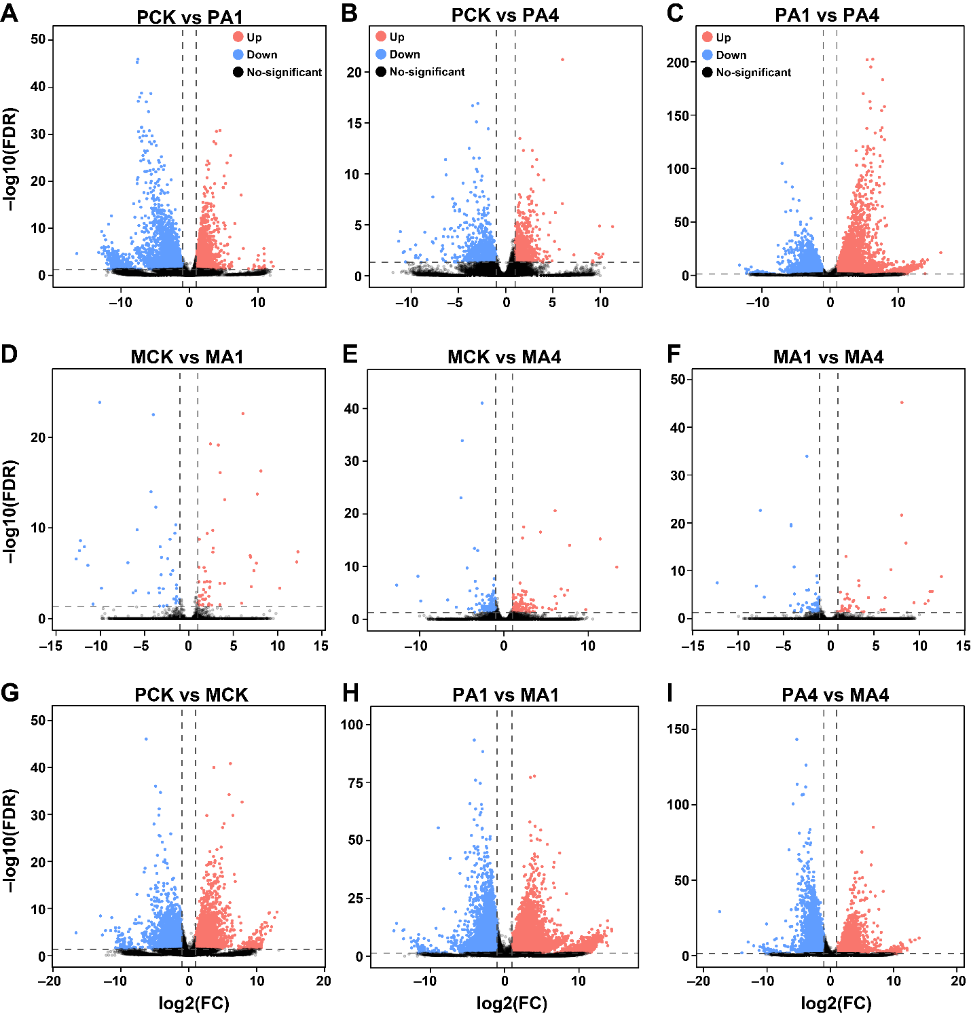
**

**Figure S2.** Volcano plots for DEGs from pair-wise comparison groups. The scattered points represent each gene in groupwise comparison; the blue dots represent down-regulated DEGs, the red dots represent up-regulated DEGs, and the black dots represent non-differentially expressed genes. Here; PCK, PA1, and PA4 represent the samples collected from plants treated with 0, 600, and 1800 kg/ha of biochar, respectively after 60 days of post-transplantation. While, MCK, MA1, and MA4 represent the samples collected from plants treated with 0, 600, and 1800 kg/ha of biochar, respectively after 100 days of post-transplantation.


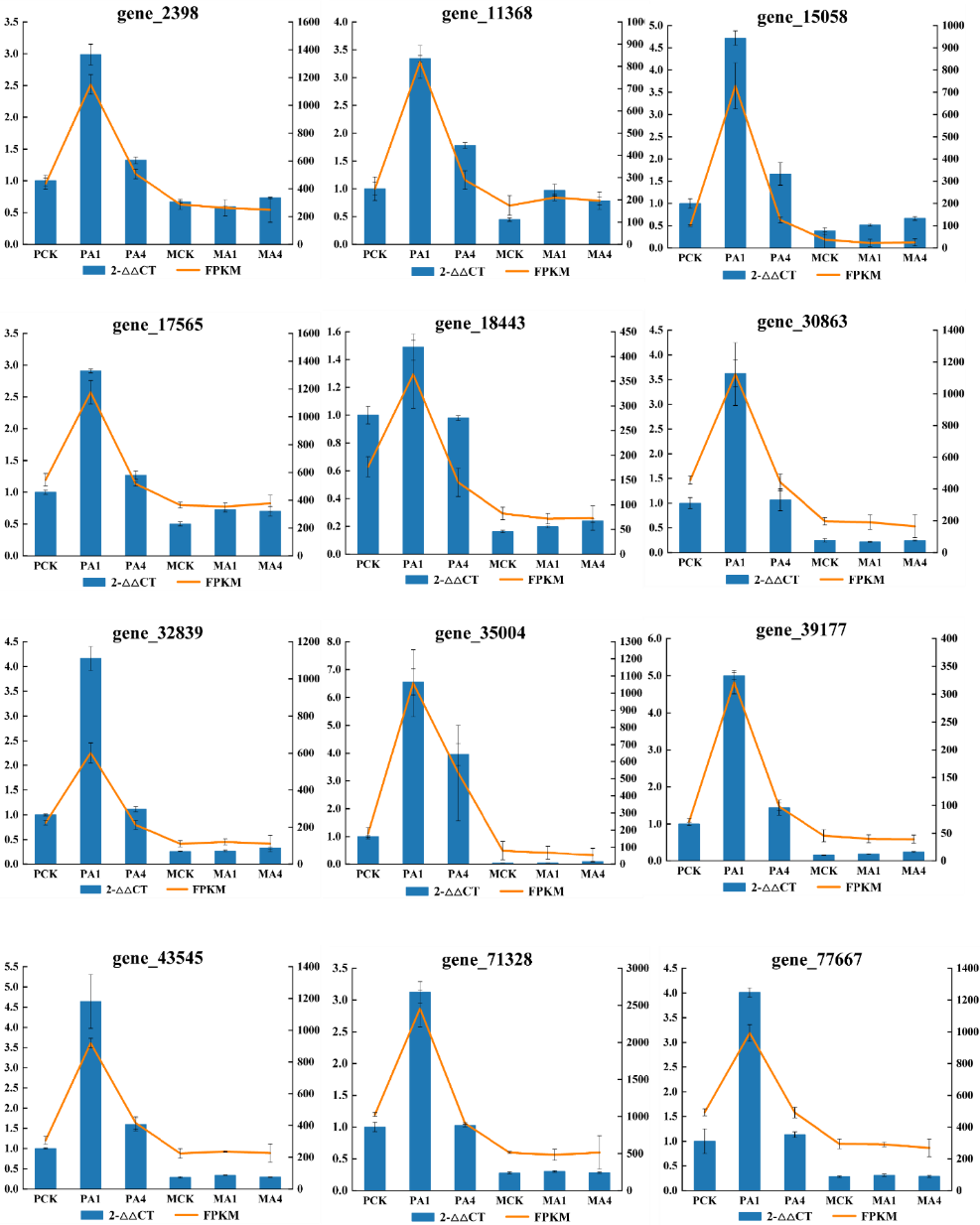


**Figure S3.** Validation of RNA-seq data by qRT-PCR. Expression levels of 12 randomly-selected DEGs from regulated pathways measured by qRT-PCR (the columns) and the corresponding expression trends recorded by RNA-Seq (the lines). The error bars represent SDs (n = 3).


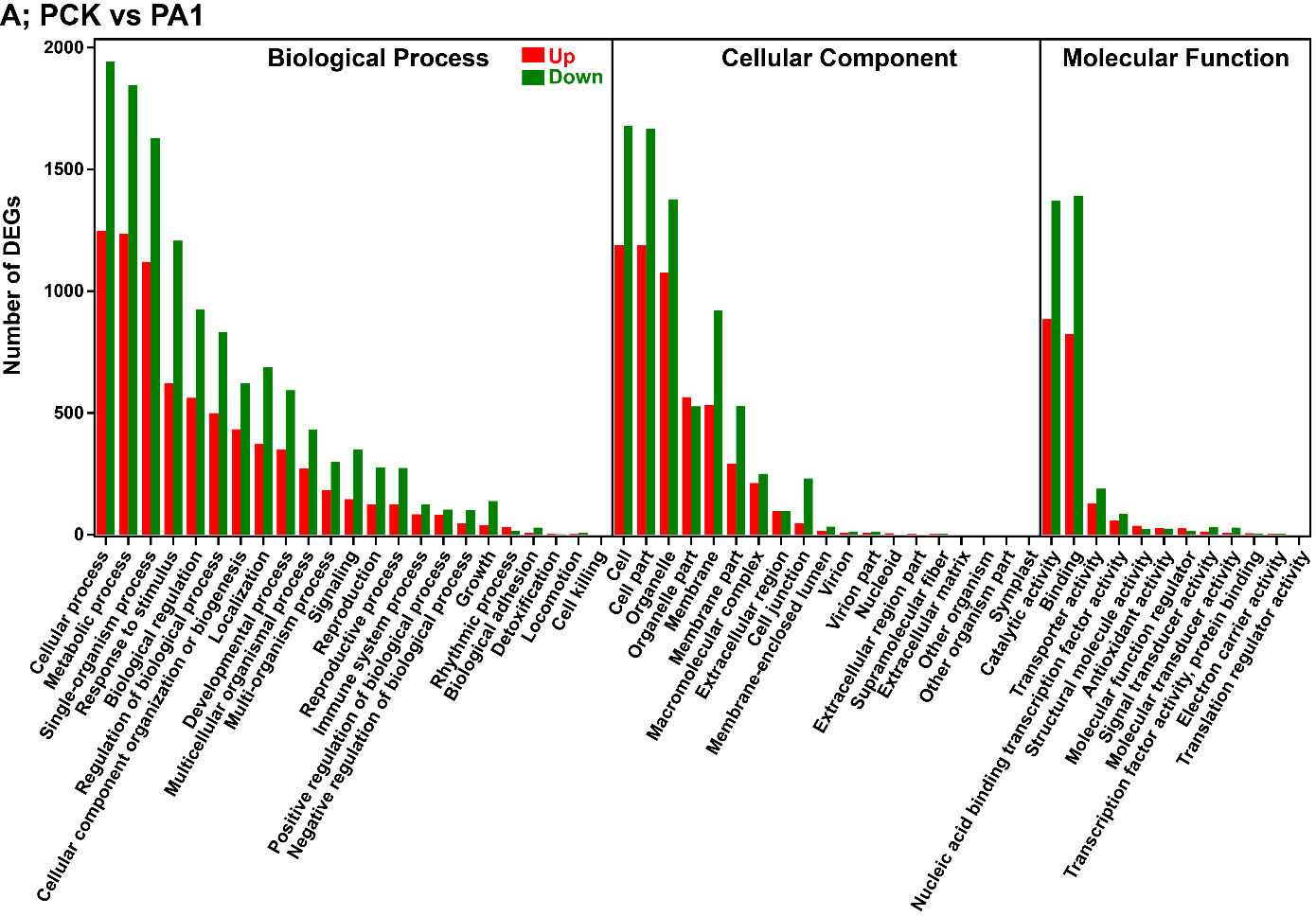


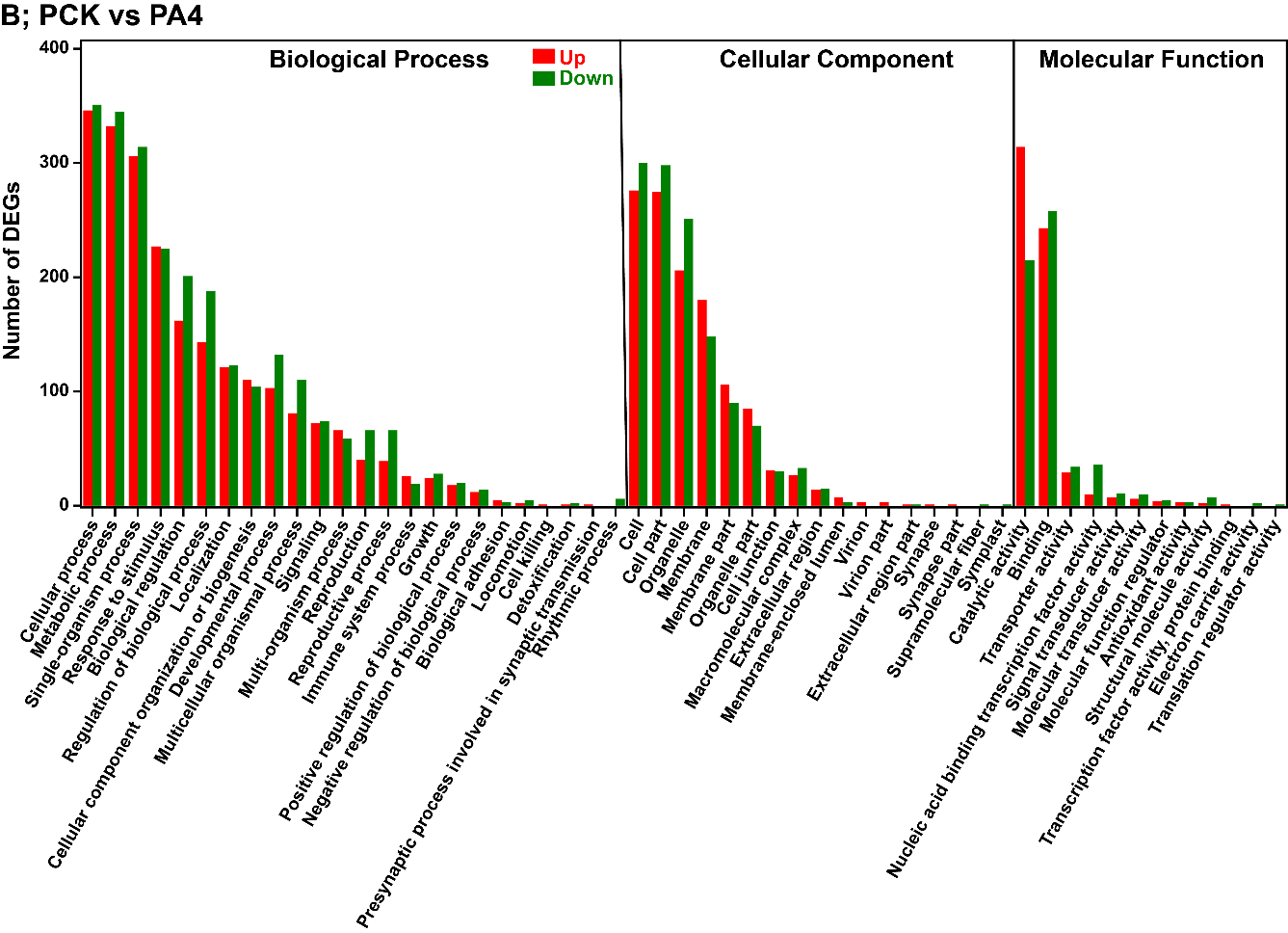


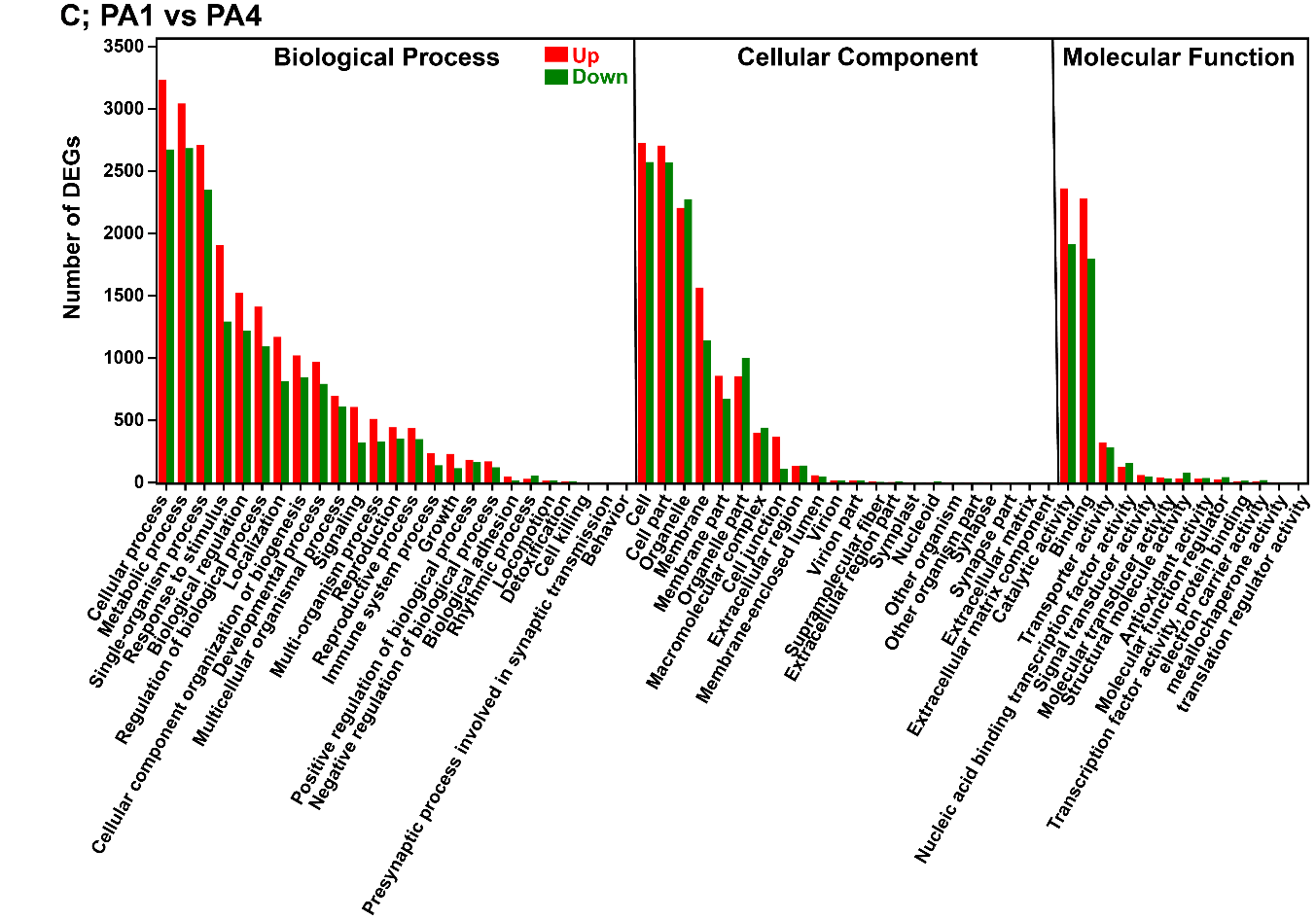


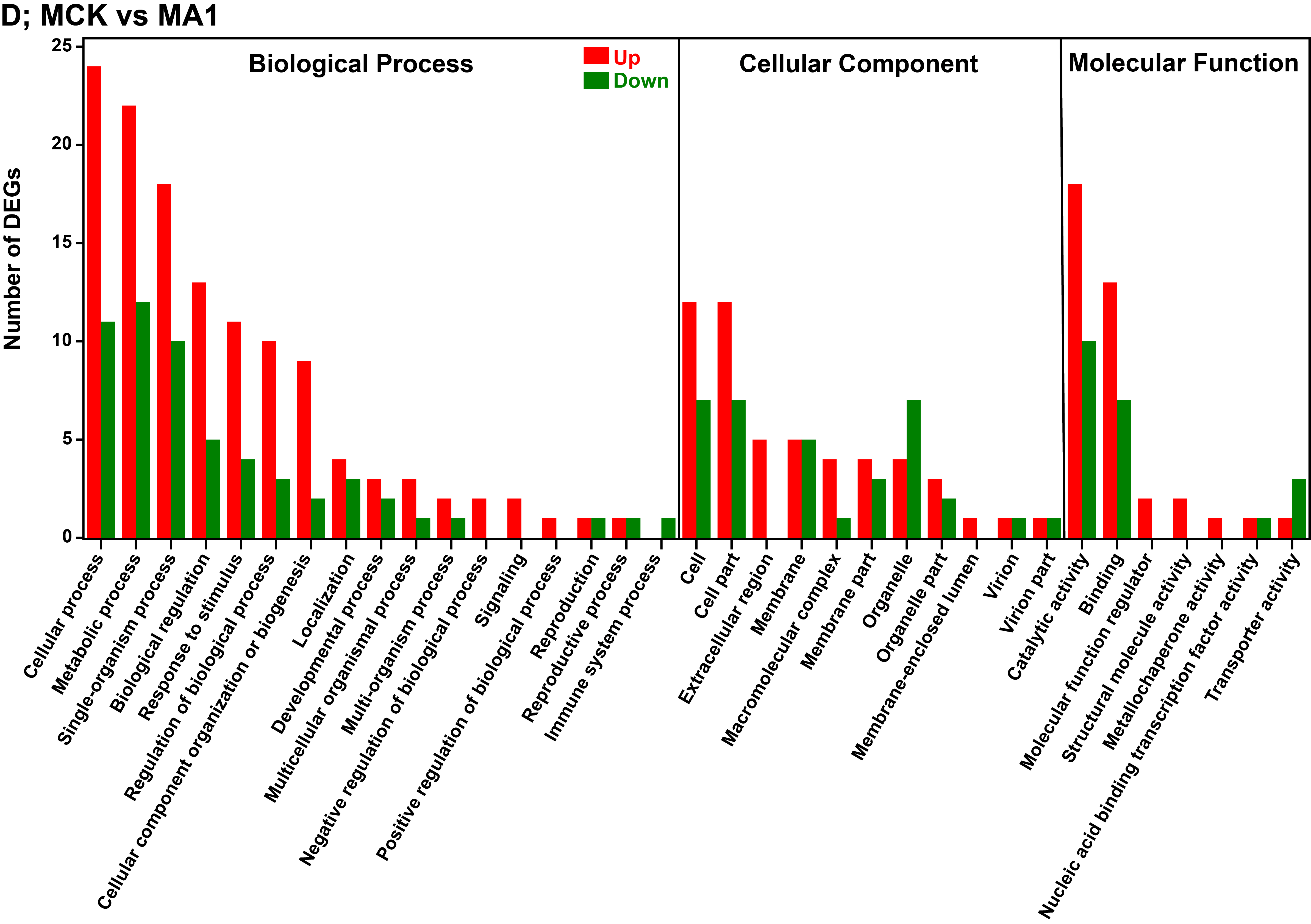


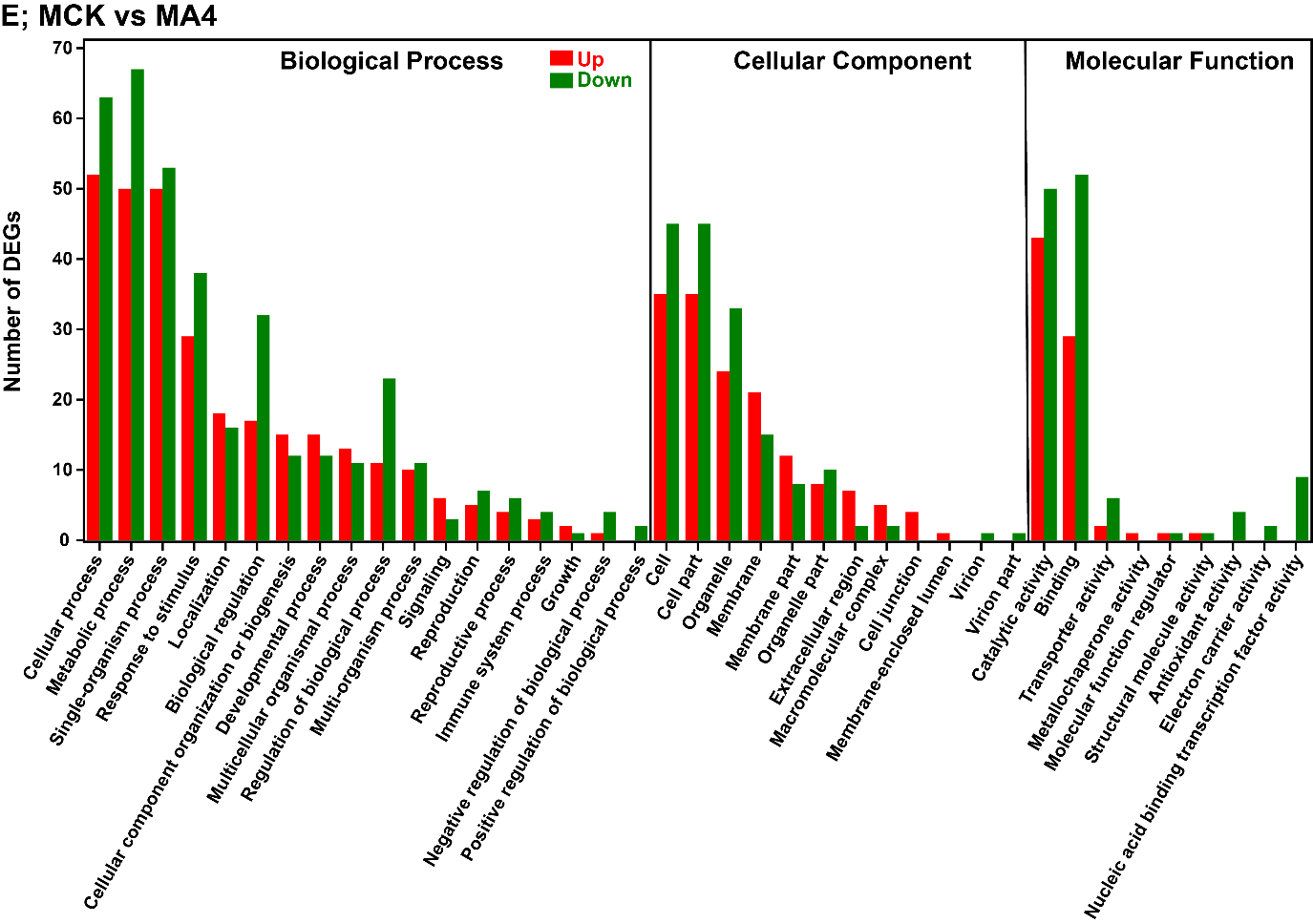


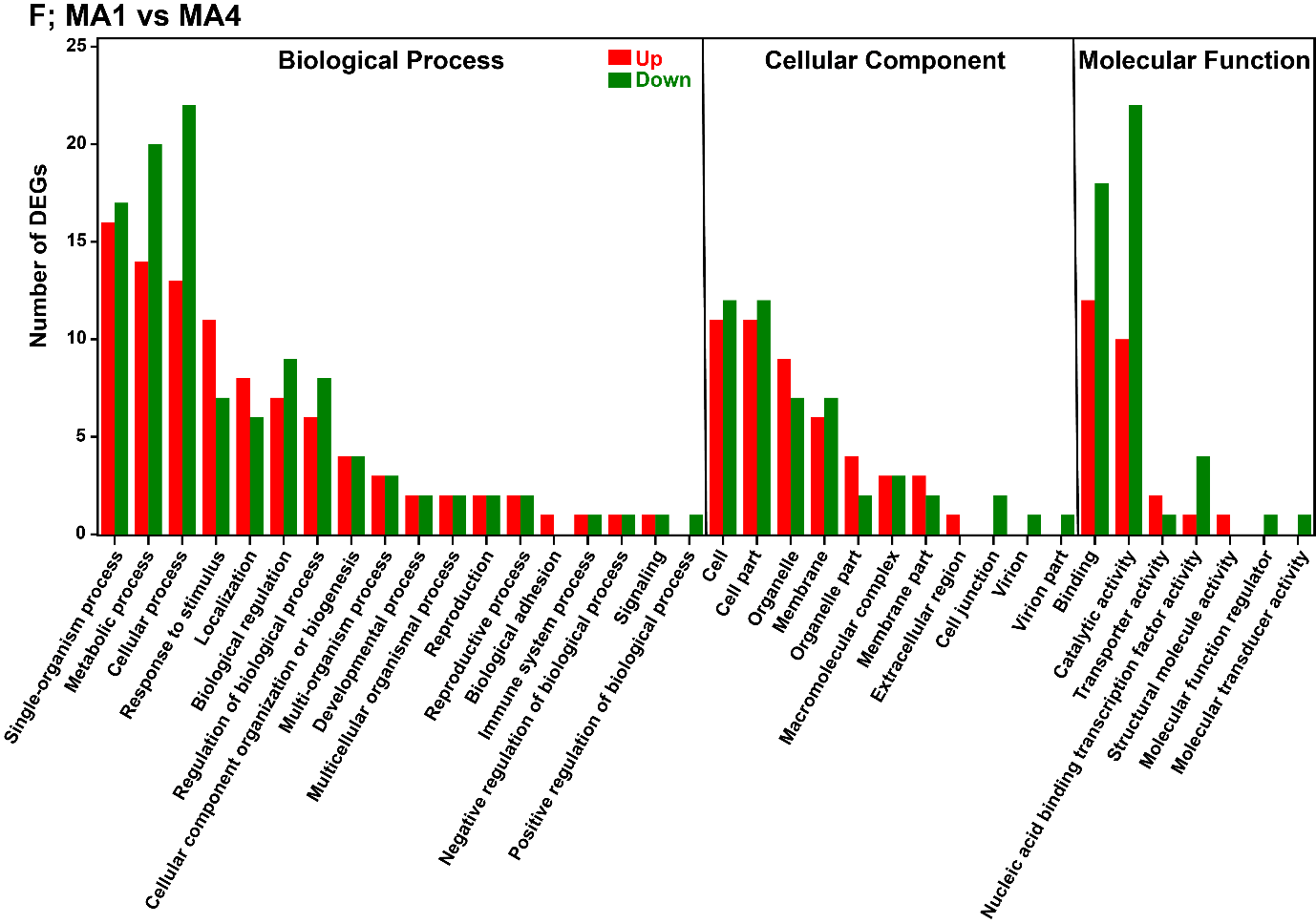


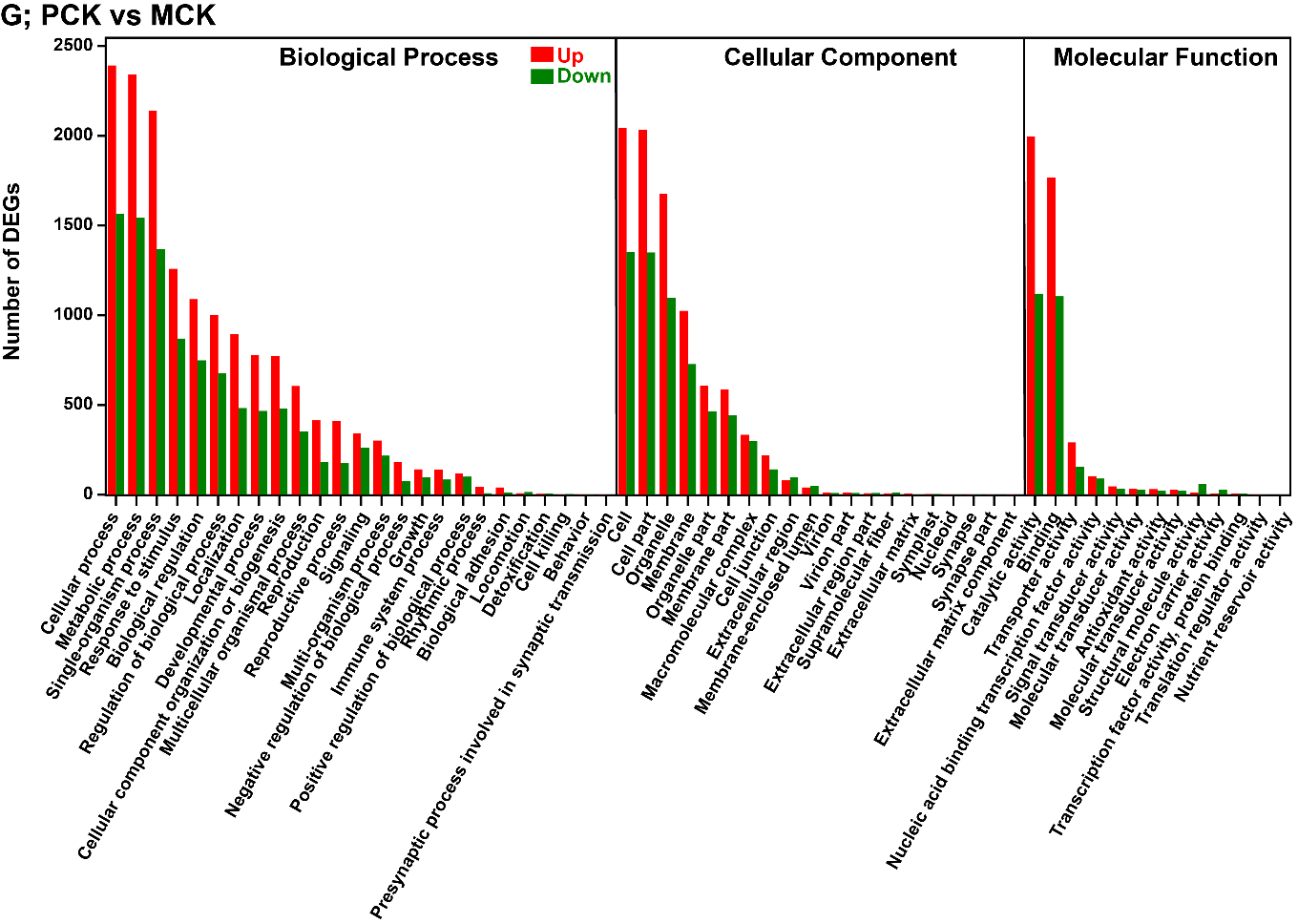


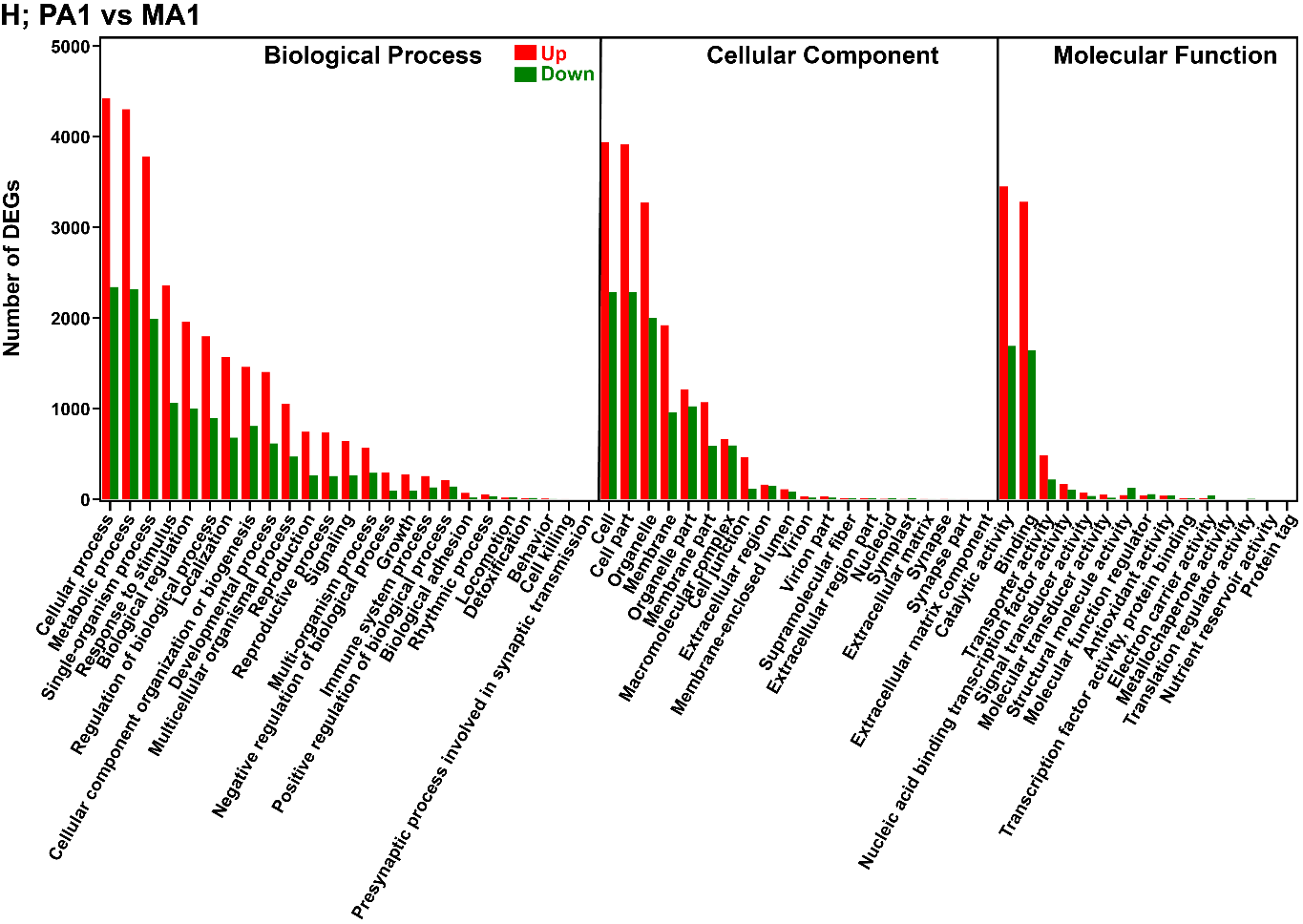


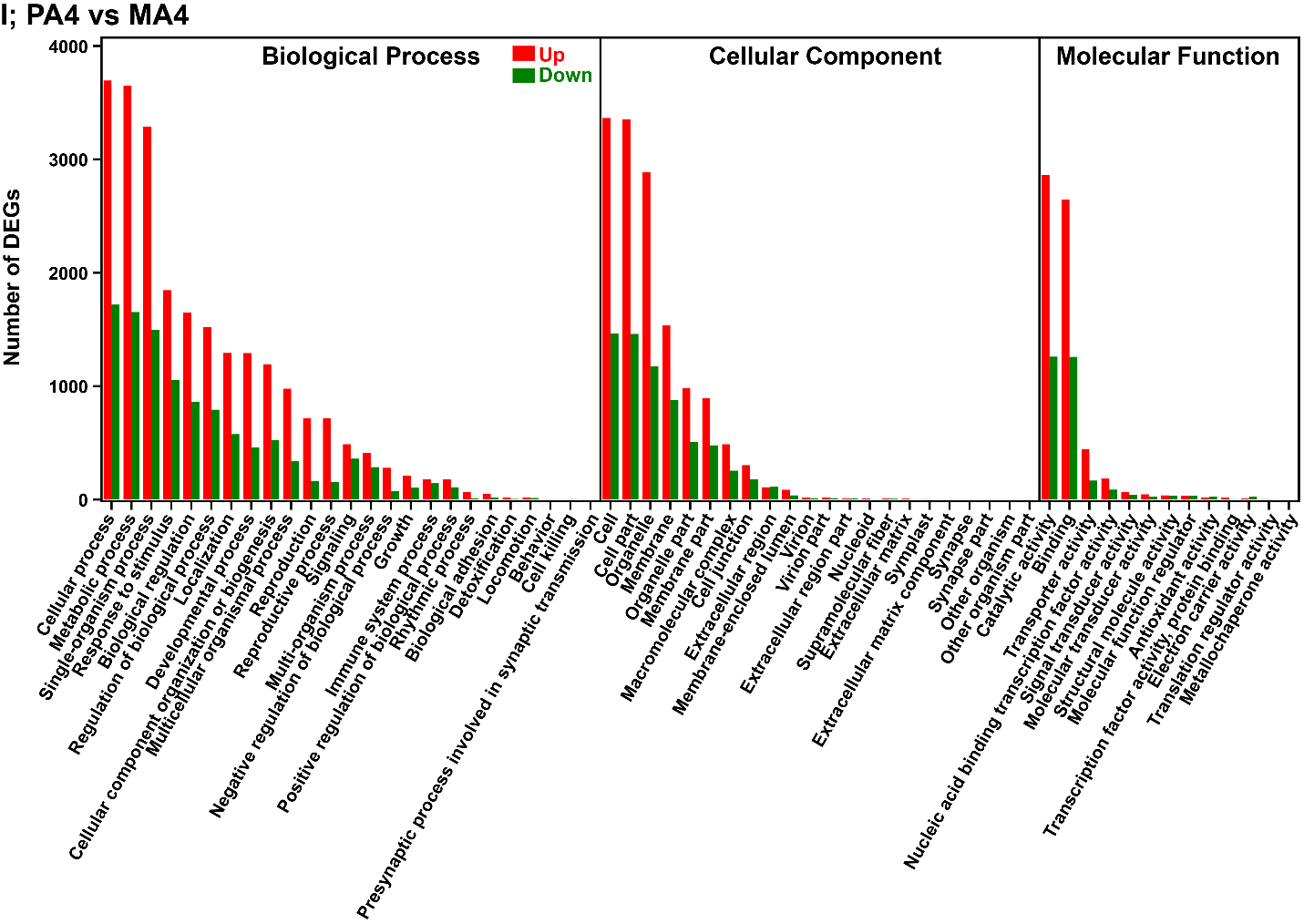


**Figure S4.** Gene Ontology (GO) term enrichment analysis. Significantly enriched GO terms were selected based on a FDR < 0.05. The Up-regulated and down-regulated DEGs from pair-wise comparison groups, in GO terms from the categories of Biological Processes, Cellular Components, and Molecular Functions are depicted in red and green, respectively. Here; PCK, PA1, and PA4 represent the samples collected from plants treated with 0, 600, and 1800 kg/ha of biochar, respectively after 60 days of post-transplantation. While, MCK, MA1, and MA4 represent the samples collected from plants treated with 0, 600, and 1800 kg/ha of biochar, respectively after 100 days of post-transplantation.

**
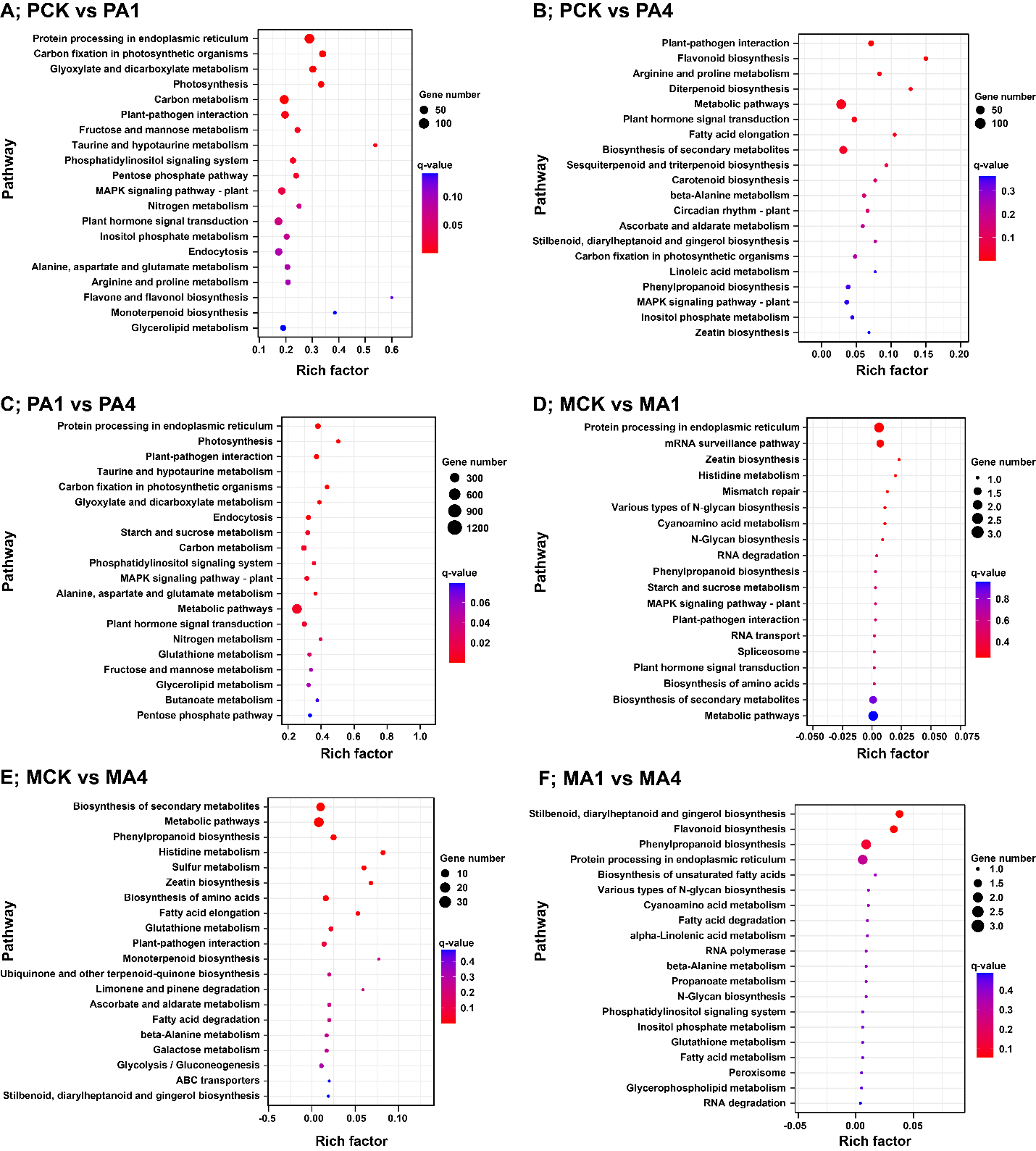
**

**
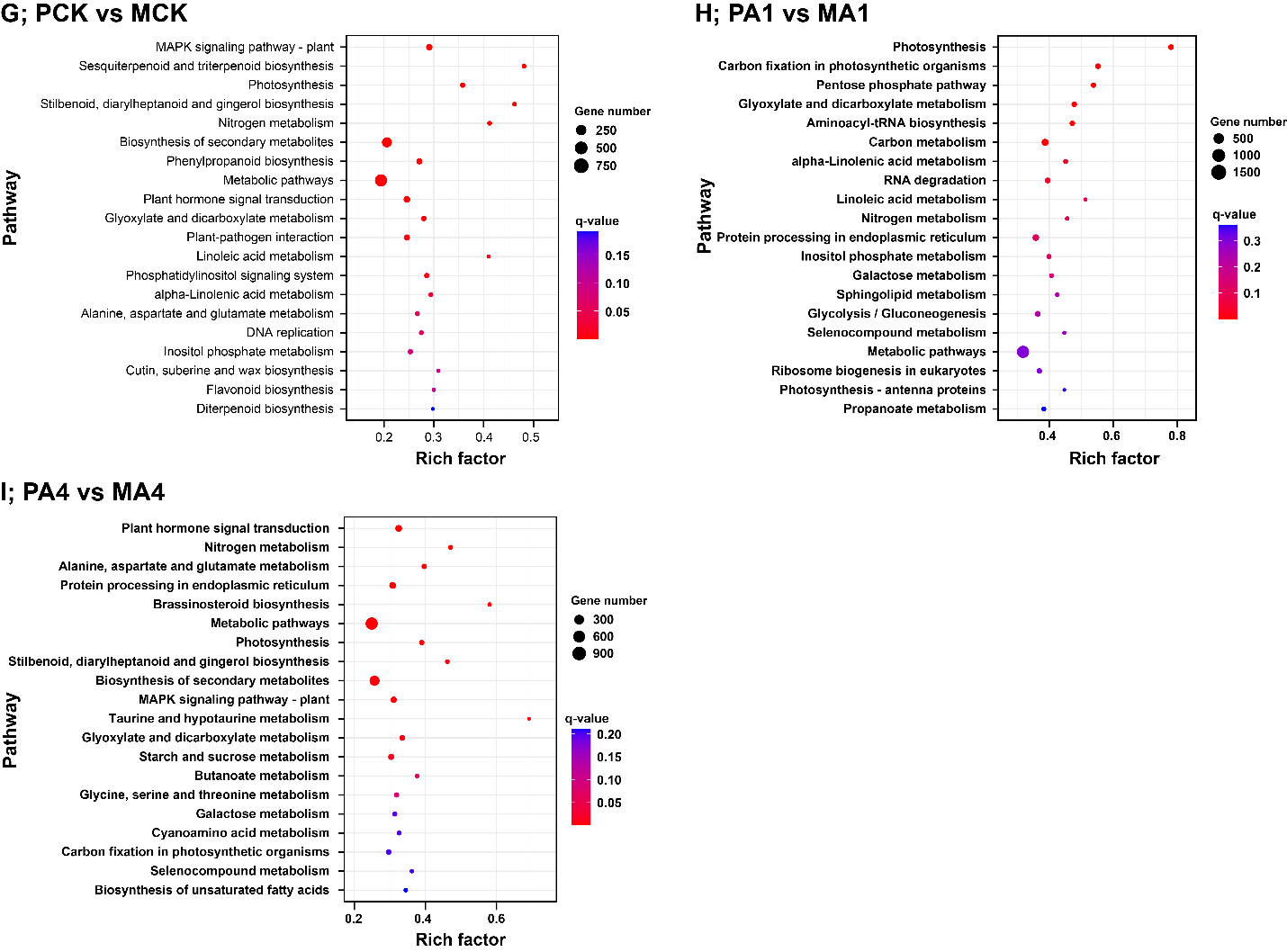
**

**Figure S5.** KEGG enrichment analysis of DEGs from pair-wise comparison groups. Each circle in the figure represents a KEGG metabolic pathway, and the size of the circle corresponds to the number of genes enriched in a pathway. The degree of significance of the enrichment of DEGs in a pathway is represented by -log10 (q-value). The abscissa indicates the ratio of the number of DEGs annotated to a particular pathway to the number of the DEGs annotated to all pathways.

**
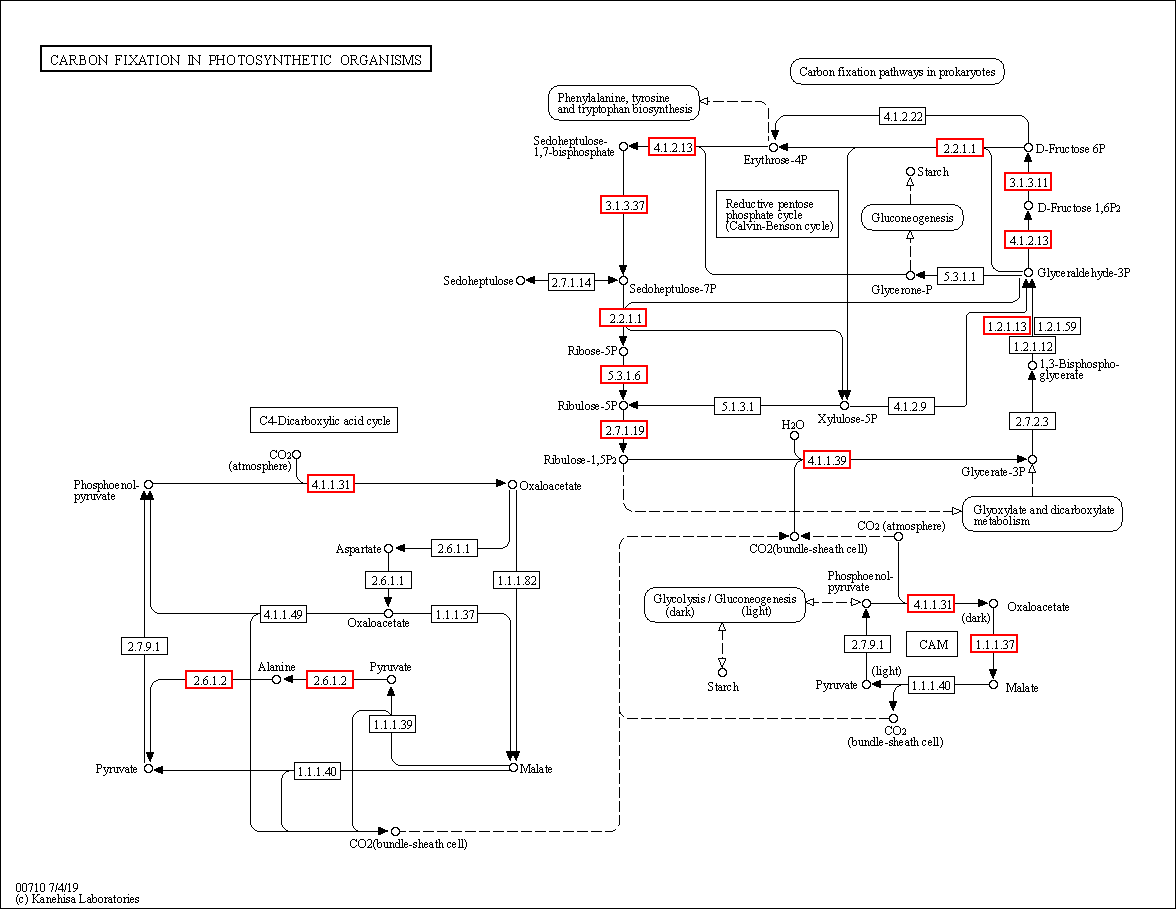
**

**Figure S6.** The carbon fixation pathway in photosynthetic organism. The red boxes highlight the upregulated transcripts in tobacco plant under 600kg/ha biochar application in groupwise PCK vs PA1 vs PA4.

**
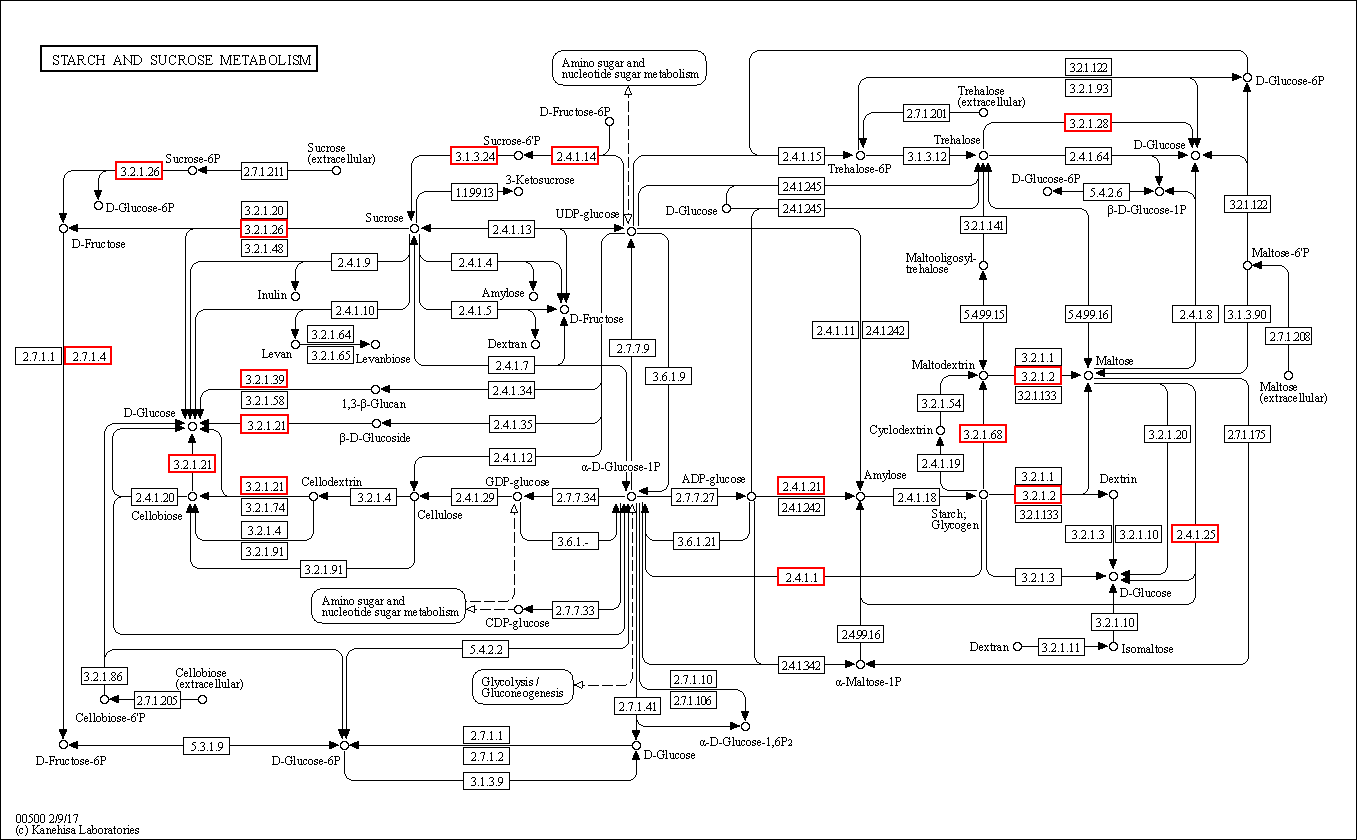
**

**Figure S7.** Starch and sucrose metabolism regulation in PCK vs PA1 vs PA4 highlight the significance of starch and sucrose balance and their role in plant growth and promotion. The red boxes show the upregulated transcripts encoding various important enzymes.
